# The risk of endocrine immune-related adverse events induced by PD-1 inhibitors in cancer patients: a systematic review and meta-analysis

**DOI:** 10.3389/fonc.2024.1381250

**Published:** 2024-05-02

**Authors:** Pengfei Zhao, Ting Zhao, Lihong Yu, Wenming Ma, Wenyu Liu, Chenning Zhang

**Affiliations:** ^1^ Department of Clinical Pharmacy, Weifang People's Hospital, Weifang, China; ^2^ Department of Pharmacy, Weifang People's Hospital, Weifang, China; ^3^ Department of Rehabilitation Medicine & Department of Pharmacy, Xiangyang No. 1 People's Hospital, Hubei University of Medicine, Xiangyang, China

**Keywords:** PD-1 inhibitors, immune-related adverse events, risk, endocrine adverse events, meta-analysis

## Abstract

**Objective:**

Endocrinopathies are the most common immune-related adverse events (irAEs) observed during therapy with PD-1 inhibitors. In this study, we conducted a comprehensive systematic review and meta-analysis to evaluate the risk of immune-related endocrinopathies in patients treated with PD-1 inhibitors.

**Methods:**

We performed a systematic search in the PubMed, Embase, and Cochrane Library databases to retrieve all randomized controlled trials (RCTs) involving PD-1 inhibitors, spanning from their inception to November 24, 2023. The comparative analysis encompassed patients undergoing chemotherapy, targeted therapy, or receiving placebo as control treatments. This study protocol has been registered with PROSPERO (CRD42023488303).

**Results:**

A total of 48 clinical trials comprising 24,514 patients were included. Compared with control groups, patients treated with PD-1 inhibitors showed an increased risk of immune-related adverse events, including hypothyroidism, hyperthyroidism, hypophysitis, thyroiditis, diabetes mellitus, and adrenal insufficiency. Pembrolizumab was associated with an increased risk of all aforementioned endocrinopathies (hypothyroidism: RR=4.76, 95%CI: 3.55-6.39; hyperthyroidism: RR=9.69, 95%CI: 6.95-13.52; hypophysitis: RR=5.47, 95%CI: 2.73-10.97; thyroiditis: RR=5.95, 95%CI: 3.02-11.72; diabetes mellitus: RR=3.60, 95%CI: 1.65-7.88; adrenal insufficiency: RR=4.80, 95%CI: 2.60-8.88). Nivolumab was associated with an increased risk of hypothyroidism (RR=7.67, 95%CI: 5.00-11.75) and hyperthyroidism (RR=9.22, 95%CI: 4.71-18.04). Tislelizumab and sintilimab were associated with an increased risk of hypothyroidism (RR=19.07, 95%CI: 5.46-66.69 for tislelizumab and RR=18.36, 95%CI: 3.58-94.21 for sintilimab). For different tumor types, both hypothyroidism and hyperthyroidism were at high risks. Besides, patients with non-small cell lung cancer were at a higher risk of thyroiditis and adrenal insufficiency. Patients with melanoma were at a higher risk of hypophysitis and diabetes mellitus. Both low- and high-dose group increased risks of hypothyroidism and hyperthyroidism.

**Conclusion:**

Risk of endocrine irAEs may vary in different PD-1 inhibitors and different tumor types. Increased awareness and understanding of the risk features of endocrine irAEs associated with PD-1 inhibitors is critical for clinicians.

**Systematic review registration:**

crd.york.ac.uk/prospero, identifier PROSPERO (CRD42023488303).

## 1 Introduction

Following surgery, radiotherapy, chemotherapy, and molecular targeted therapy, immunotherapy represents another important method in cancer treatment. Among these, immune checkpoint inhibitors (ICPIs) are the most promising. In contrast to conventional cancer treatments, ICPIs are monoclonal antibodies that specifically target the inhibitory receptors on T cells. They work by blocking the negative regulatory factors that impede T cell activity, thereby activating T cells and harnessing the body’s innate immune response to fight cancer. They systematically bolster the body’s immune response against tumors, resulting in a marked enhancement of cancer patients’ overall survival rates (1–3). Presently, immune checkpoint inhibitors, with programmed death-1 (PD-1) inhibitors as a prominent example, have shown significant therapeutic effects in the treatment of various malignant tumors, benefiting an increasing number of cancer patients.

While ICPIs activate the immune system to target cancer cells, they also downregulate tolerance to self-antigens, which may lead to immune-related adverse events (irAEs) in normal tissues. IrAEs can affect almost any tissue or organ in the human body. According to the literature, nearly two-thirds of patients treated with immune checkpoint inhibitor experienced irAEs of varying degrees, which can occur at any point during therapy (4, 5). Most of these irAEs are generally mild, and with early identification and intervention, most of them can be reversed. However, there is still a range of 0.5% to 13.0% of patients with severe irAEs (6, 7). Although the severe irAEs are uncommon, once they occur, the consequences can be serious, and in some cases, even fatal.

Endocrine system irAEs are one of the most common adverse reactions associated with PD-1 inhibitors, mainly affecting endocrine glands including the pituitary, thyroid, pancreas, and adrenal glands (8, 9). Among them, hypothyroidism, hyperthyroidism, and pituitary inflammation are relatively common, with incidence rates of 11%, 4%, and 1%, respectively (10). Additionally, there have also been reports of primary adrenal insufficiency and autoimmune diabetes (11, 12). The onset time for endocrine irAEs varies widely, but they typically manifest slowly and are often delayed. The median time to onset is 9 weeks after the start of treatment, with a range from 5 to 36 weeks (13). These endocrine disorders often lack specific clinical symptoms, with most patients only noticing changes in relevant biochemical markers, making diagnosis challenging. Many endocrine function abnormalities may not recover, potentially endangering patients’ lives if not promptly identified and treated. We conducted this systematic review and meta-analysis to evaluate the risks of endocrine-related adverse events in cancer patients treated with PD-1 inhibitors, compared to those receiving control treatments.

## 2 Methods

This systematic review and meta-analysis were performed in accordance with the Cochrane Handbook for Systematic Reviews of Interventions (14) and followed the Preferred Reporting Items for Systematic Reviews and Meta-Analyses (PRISMA) guidelines (15). The study protocol was prospectively registered on PROSPERO, the International Prospective Register of Systematic Reviews (accessible at crd.york.ac.uk/prospero, Identifier: CRD42023488303). In China, the conduction of this meta-analysis did not necessitate the approval of a formal research ethics committee.

### 2.1 Search strategy

The literature search was conducted following the PICOS framework, encompassing patient characteristics, interventions, comparisons, outcomes, and study designs. We systematically searched databases including PubMed, Embase, and the Cochrane Library for randomized controlled trials (RCTs) from their inception until November 24, 2023. To illustrate with PubMed, the following terms were used to retrieve relevant studies: (pembrolizumab[Title/Abstract] OR nivolumab[Title/Abstract] OR camrelizumab[Title/Abstract] OR toripalimab[Title/Abstract] OR tislelizumab[Title/Abstract] OR sintilimab[Title/Abstract]) AND (randomized controlled trial[Publication Type] OR randomized[Title/Abstract] OR placebo[Title/Abstract]). The search strategy was specifically adjusted for each database, as detailed in **Supplementary Table S1**. The search was restricted to RCTs published in English. Additionally, we backtracked the references cited by the identified studies to further expand our pool of eligible studies. The retrieval process was organized using the bibliographic management software EndNote X9.

### 2.2 Study selection and eligibility criteria

The aim of this study was to assess the relative risks of endocrine irAEs associated with PD-1 inhibitors. Study inclusion was based on the fulfillment of predefined criteria: (1) they must be randomized controlled clinical trials; (2) they should involve patients diagnosed with any type of malignancy, without restrictions on cancer type, sex, age, or geographical location; (3) patients must be randomly allocated to either PD-1 inhibitor monotherapy or control treatments, such as chemotherapy, targeted drugs, or placebo. Previous oncologic treatments before the initiation of PD-1 inhibitor therapy were considered acceptable; (4) studies must provide available data on endocrine-related adverse events.

Studies that failed to meet the selection criteria were systematically excluded. Additional exclusion criteria included: (1) phase I trials, duplicate publications, single-arm cohort studies, unpublished manuscripts, meeting abstracts and retrospective analyses; (2) studies based on animal models or *in vitro* cell lines; (3) trials in which patients in the intervention groups were treated with PD-1 inhibitors in conjunction with chemotherapy, radiotherapy, or any other concurrent therapies.

### 2.3 Data extraction and quality assessment

Two independent researchers conducted the literature screening, data extraction, and quality assessment of the studies. Using the specified inclusion and exclusion criteria, the initial selection of studies was made based on titles and abstracts to ascertain their adherence to the criteria. Following the selection, full-text articles were retrieved, downloaded, and organized in EndNote X9 for reference management. A bespoke standardized data collection table was utilized to record relevant information from each included clinical trial.

The extracted data included: (1) patient demographics, including the average age, gender distribution, tumor types, specific PD-1 inhibitors used, and dosing regimens; (2) study details, such as the first author’s name, publication year, NCT identification number, trial phase, descriptions of the treatment and control arms, and the total number of patients treated with PD-1 inhibitors versus those receiving control treatments; (3) outcome data, detailing the types of irAEs (hypothyroidism, hyperthyroidism, hypophysitis, thyroiditis, diabetes mellitus, and adrenal insufficiency) and the number of patients experiencing all-grade immune-related endocrinopathies. Data from each study was scrutinized repeatedly to guarantee thorough and accurate extraction.

The quality of the included randomized controlled trials was evaluated using the Cochrane Collaboration’s Risk of Bias Tool, as outlined in the Handbook for Systematic Reviews of Interventions. This assessment included seven domains: random sequence generation (to assess selection bias), allocation concealment (to assess selection bias), blinding of participants and personnel (to assess performance bias), blinding of outcome assessment (to assess detection bias), completeness of outcome data (to assess attrition bias), selective reporting of outcomes (to assess reporting bias), and the presence of other potential sources of bias. Each domain was evaluated and classified based on the level of risk: ‘high risk’ was denoted with red, ‘low risk’ with green, and ‘unclear risk’ with yellow in the corresponding bias graphs. Any discrepancies encountered during the evaluation were resolved through discussions until a consensus was achieved.

### 2.4 Statistical analysis

Statistical analyses were performed using Review Manager 5.3 (Cochrane Collaboration, Copenhagen, Denmark) and STATA 14.0 (STATA Corporation, USA). For each included study, the relative risk (RR) and 95% confidence intervals (95%CI) were calculated to evaluate the risk of endocrine disorders. An RR exceeding 1.0 indicated an increased risk of endocrine-related adverse events in patients treated with PD-1 inhibitors compared to those receiving control treatments.

We selected the appropriate model, either fixed-effect or random-effect, based on the heterogeneity of the studies, as indicated by the Cochran Q statistic and I^2^ index. Heterogeneity was considered significant when the *p*-value was below 0.1. I^2^ values of less than 50%, between 50-75%, and above 75% indicated low, moderate, and high heterogeneity, respectively. A random-effect model was employed in the presence of significant heterogeneity, while a fixed-effect model was selected when heterogeneity was not significant. The Mantel-Haenszel method was used to combine the individual study risk ratios into an integrative forest plot.

*P*-values were calculated as two-tailed, with a threshold of less than 0.05 denoting statistical significance. In cases where an RCT included multiple intervention arms, each was individually compared to the control arm. Subgroup analyses were performed to explore differences based on endocrinopathies, tumor types, specific PD-1 inhibitors, dosages, and prior treatments. Finally, funnel plots were employed to assess potential publication bias.

## 3 Results

### 3.1 Eligible studies

**Figure 1:**
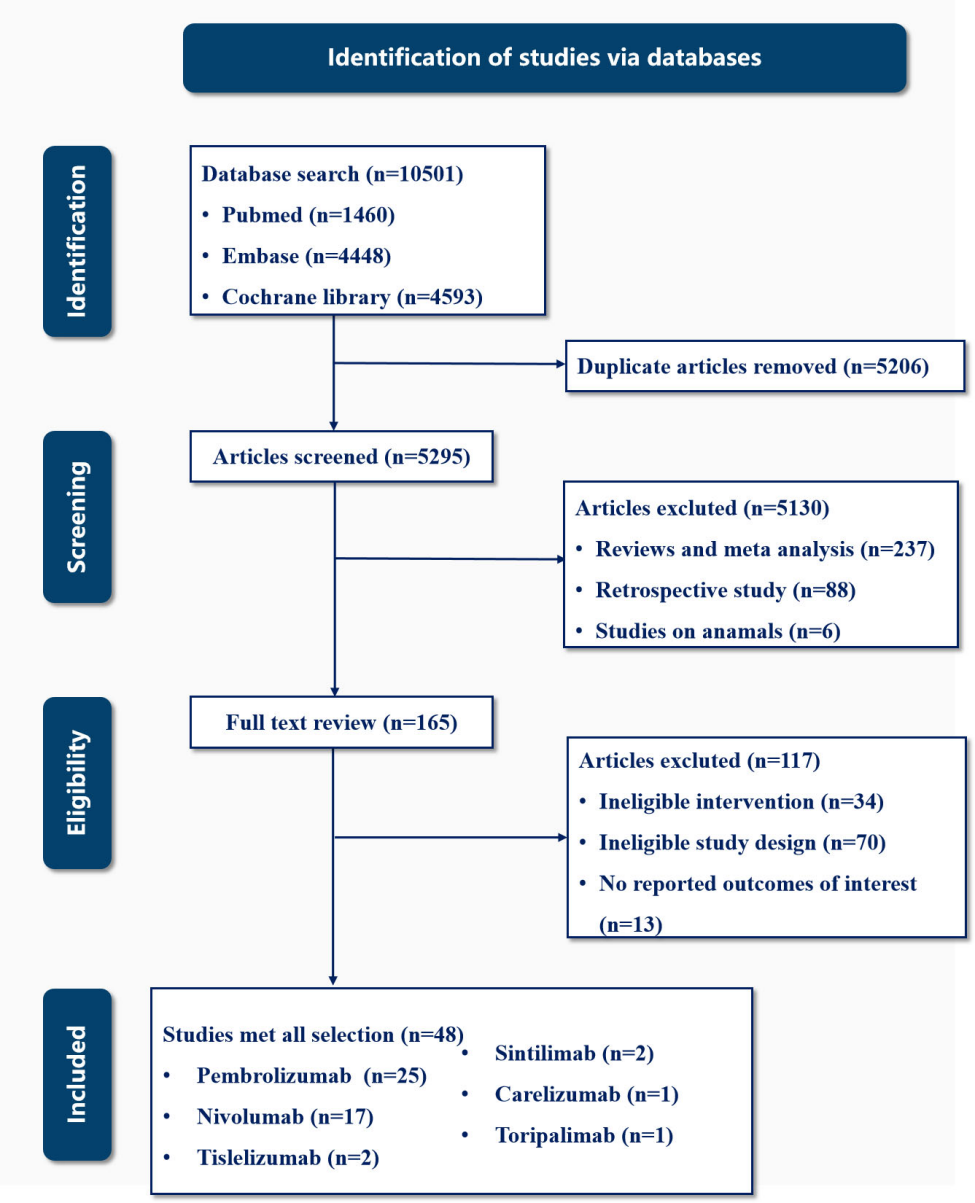
Flowchart of the study selection process.

We initially identified 10,501 potentially relevant clinical studies–1,460 from PubMed, 4,448 from Embase, and 4,593 from the Cochrane Library. Upon full-text review, 48 studies were finally included (16–63). These studies illustrated the immune-related endocrinopathies associated with PD-1 inhibitors, involving a collective patient cohort of 24,514 (13,121 in the intervention arm and 11,393 in the control arm). The selection process was showed in **Figure 1**, and **Supplementary Figures S1**, **S2** presented the quality assessment of the included trials. The symmetry observed in the funnel plots indicated no detectable publication bias.

### 3.2 Study characteristics

Involved PD-1 inhibitors included nivolumab (17 trials including 8153 patients), pembrolizumab (25 trials including 14 026 patients), camrelizumab (1 trials including 448 patients), toripalimab (1 trials including 145 patients), tislelizumab (2 trials including 1287 patients) and sintilimab (2 trials including 455 patients). The majority of the RCTs (34 trials) assessed PD-1 inhibitors in comparison to chemotherapy, while 3 trials compared them with targeted drugs, 12 with placebo, and 1 with interferon used as monotherapy. Endocrine irAEs were closely monitored, with hypothyroidism observed in 49 trials including 23,784 patients, hyperthyroidism in 37 trials including 18,060 patients, hypophysitis in 22 trials including 12,172 patients, thyroiditis in 19 trials including 10,889 patients, diabetes mellitus in 19 trials including 10,760 patients, and adrenal insufficiency in 21 trials including 12,388 patients. The baseline characteristics of the 48 RCTs were detailed in **Supplementary Tables S2**-**S7**. **Table 1** showed the statistically significant results regarding the relative risks of immune-related endocrine adverse events associated with PD-1 inhibitors.

**Table 1:** Relative risks of immune-related endocrine adverse events caused by PD-1 inhibitors.

Subgroup	Groups	Hypothyroidism	Hyperthyroidism	Thyroiditis	Hypophysitis	Adrenal insufficiency	Diabetes mellitus
Types of PD-1 inhibitors	Pembrolizumab	4.76 (3.55, 6.39)	9.69 (6.95, 13.52)	5.95 (3.02, 11.72)	5.47 (2.73, 10.97)	4.80 (2.60, 8.88)	3.60 (1.65, 7.88)
	Nivolumab	7.67 (5.00, 11.75)	9.22 (4.71, 18.04)	NS	NS	NS	NS
	Tislelizumab	19.07 (5.46, 66.69)	NR	NR	NR	NR	NR
	Sintilimab	18.36 (3.58, 94.21)	NR	NR	NR	NR	NR
	Camrelizumab	NR	NR	NR	NR	NR	NR
	Toripalimab	NR	NR	NR	NR	NR	NR
Tumor types	NSCLC	10.51 (6.97, 15.87)	8.33 (4.80, 14.44)	5.47 (1.41, 21.13)	NS	4.55 (1.15, 17.97)	NS
	Melanoma	4.82 (2.73, 8.52)	11.15 (6.41, 19.40)	NS	8.26 (2.75, 24.84)	NS	5.38 (1.20, 24.12)
	HNSCC	3.26 (2.18, 4.88)	3.00 (1.05, 8.52)	NR	NS	NS	NR
	Digestive system tumor	6.24 (3.80, 10.25)	10.50 (4.54, 24.26)	NS	NS	NS	NS
Dosage	Low	5.49 (4.25, 7.09)	10.33 (7.63, 14.01)	NR	4.95 (2.60, 9.42)	NR	NR
	High	19.08 (4.64, 78.53)	5.83 (1.91, 17.85)	NR	NS	NR	NR
Previous treatment	Yes	5.85 (4.27, 8.02)	8.86 (6.26, 12.55)	3.73 (1.84, 7.56)	3.97 (1.84, 8.57)	3.02 (1.52, 6.01)	2.23 (1.07, 4.64)
	No	5.55 (3.50, 8.79)	13.10 (7.57, 22.66)	6.72 (2.50, 18.08)	6.42 (2.25, 18.33)	7.81 (2.92, 20.84)	4.99 (1.45, 17.17)

HNSCC, Head-and-neck squamous cell carcinoma; NSCLC, Non-small cell lung cancer; NR, Not reported; NS, No significance.

### 3.3 Subgroup analysis by different PD-1 inhibitors

**Figure 2:**
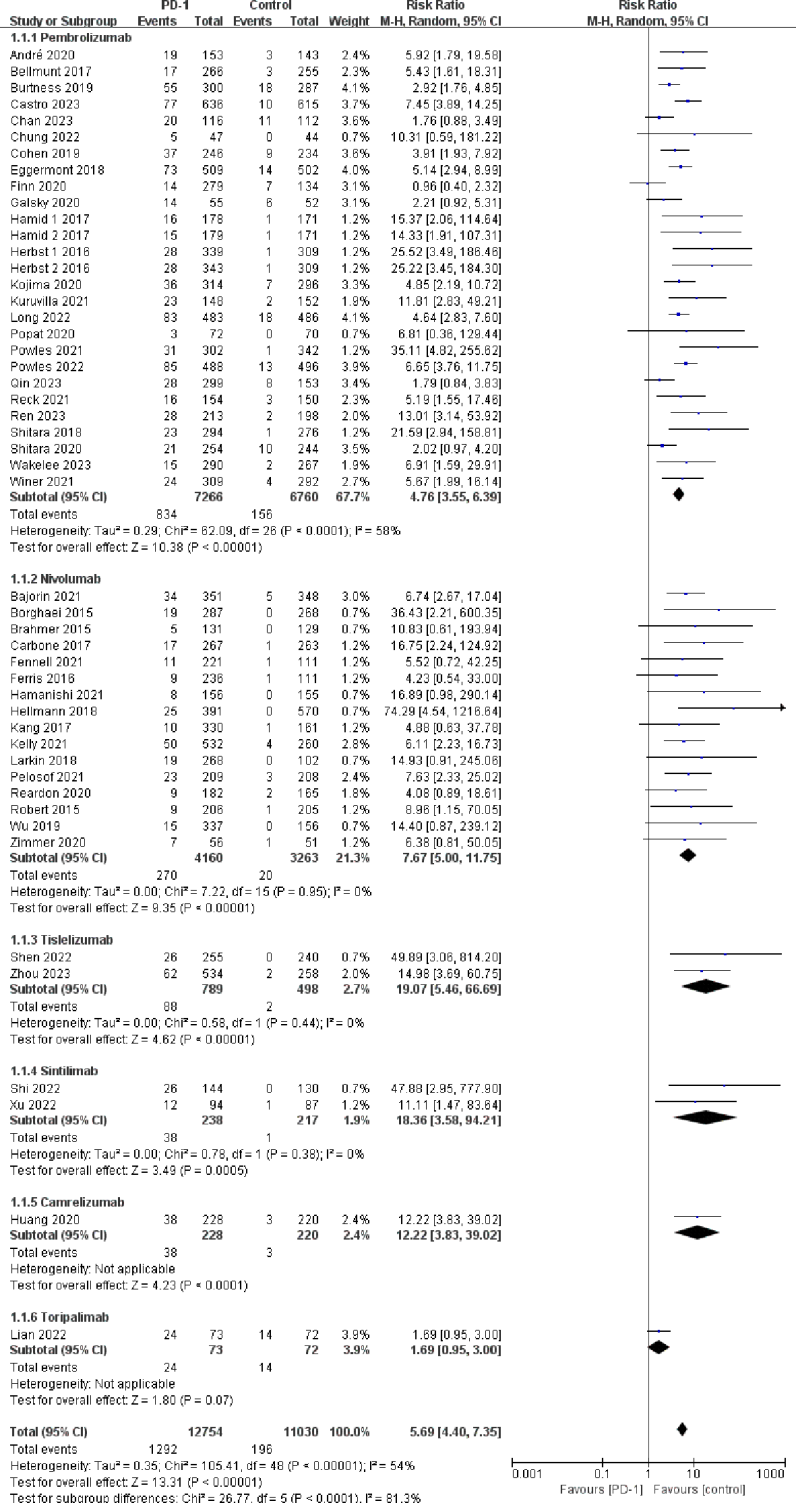
Forest plots of the relative risks of hypothyroidism related to different PD-1 inhibitors.

**Figure 3:**
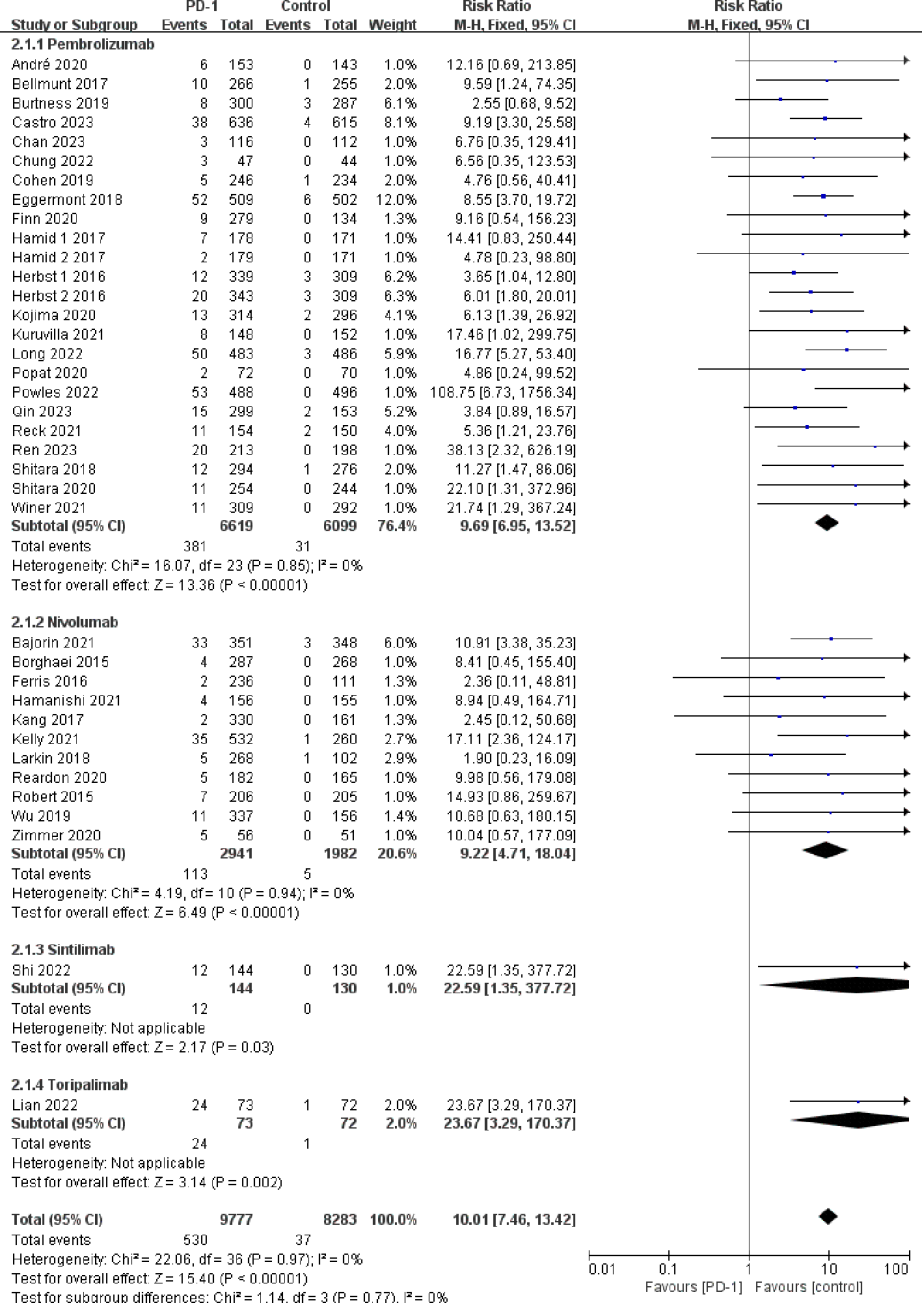
Forest plots of the relative risks of hyperthyroidism related to different PD-1 inhibitors.

**Figure 4:**
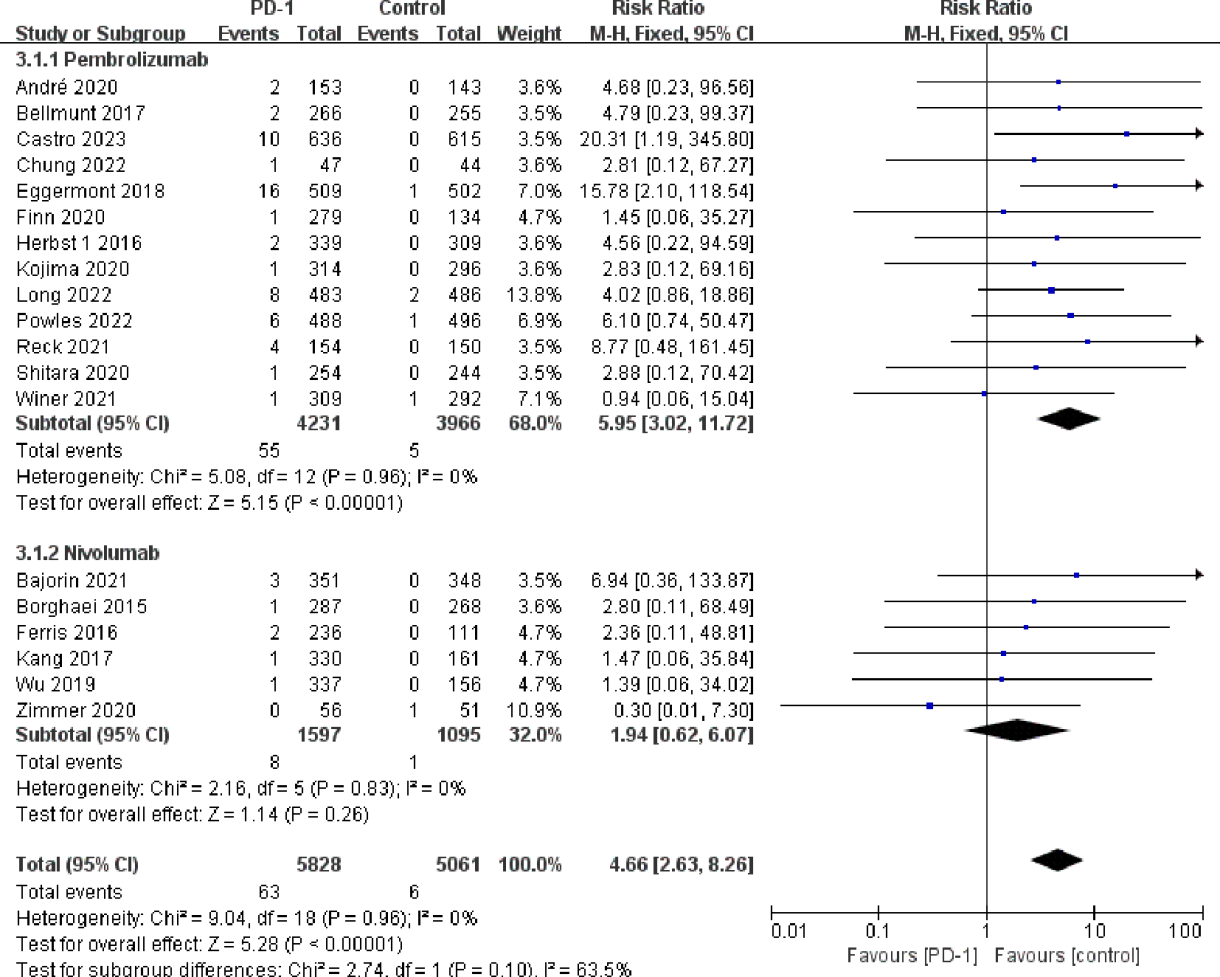
Forest plots of the relative risks of thyroiditis related to different PD-1 inhibitors.

Compared with the control groups, patients treated with PD-1 inhibitors exhibited a significantly increased risk of hypothyroidism (RR=5.69, 95%CI: 4.40-7.35) (**Figure 2**), hyperthyroidism (RR=10.01, 95%CI: 7.46-13.42) (**Figure 3**), thyroiditis (RR=4.66, 95%CI: 2.63-8.26) (**Figure 4**), hypophysitis (RR=4.77, 95%CI: 2.57-8.84) (**Supplementary Figure S3**), adrenal insufficiency (RR=4.40, 95%CI: 2.53-7.65) (**Supplementary Figure S4**) and diabetes mellitus (RR=2.85, 95%CI: 1.53-5.31) (**Supplementary Figure S5**).

Pembrolizumab was associated with a significantly increased risk of endocrine adverse events, including hypothyroidism (RR=4.76, 95%CI: 3.55-6.39), hyperthyroidism (RR=9.69, 95%CI: 6.95-13.52), thyroiditis (RR=5.95, 95%CI: 3.02-11.72), hypophysitis (RR=5.47, 95%CI: 2.73-10.97), diabetes mellitus (RR=3.60, 95%CI: 1.65-7.88), and adrenal insufficiency (RR=4.80, 95%CI: 2.60-8.88). Nivolumab was associated with increased risk of hypothyroidism (RR=7.67, 95%CI: 5.00-11.75) and hyperthyroidism (RR=9.22, 95%CI: 4.71-18.04), but it did not exhibit a statistically significant increase in the risk of thyroiditis (RR=1.94, 95%CI: 0.62-6.07), hypophysitis (RR=2.44, 95%CI: 0.60-9.90), diabetes mellitus (RR=1.62, 95%CI: 0.52-5.06), and adrenal insufficiency (RR=2.79, 95%CI: 0.68-11.37). Both tislelizumab and sintilimab were associated with an increased risk of hypothyroidism (RR=19.07, 95%CI: 5.46-66.69 for tislelizumab and RR=18.36, 95%CI: 3.58-94.21 for sintilimab) (**Figures 2**–**4**; **Supplementary Figures S3**-**S5**).

### 3.4 Subgroup analysis by tumor types

Analyzing the types of cancer involved in clinical trials, 13 studies focused on non-small cell lung cancer (NSCLC), 8 on melanoma, 3 on head-and-neck squamous cell carcinoma (HNSCC), and 11 on digestive system tumor. As showed in **Supplementary Figures S6**-**S11**, patients with NSCLC exhibited an increased risk for hypothyroidism (RR=10.51, 95%CI: 6.97-15.87), hyperthyroidism (RR=8.33, 95%CI: 4.80-14.44), thyroiditis (RR=5.47, 95%CI: 1.41-21.13), and adrenal insufficiency (RR=4.55, 95%CI: 1.15-17.97), but the increased risk for hypophysitis (RR=3.77, 95%CI: 0.80-17.65) and diabetes mellitus (RR=1.77, 95%CI: 0.50-6.31) were not statistically significant. Patients with melanoma had an increased risk for hypothyroidism (RR=4.82, 95%CI: 2.73-8.52), hyperthyroidism (RR=11.15, 95%CI: 6.41-19.40), hypophysitis (RR=8.26, 95%CI: 2.75-24.84), and diabetes mellitus (RR=5.38, 95%CI: 1.20-24.12). However, no significant increase was observed in the risk for thyroiditis (RR=3.74, 95%CI: 0.60-23.18) and adrenal insufficiency (RR=4.73, 95%CI: 0.15-146.22). For patients with HNSCC and digestive system tumors, an increased risk was noted for hypothyroidism (RR=3.26, 95%CI: 2.18-4.88 for HNSCC and RR=6.24, 95%CI: 3.80-10.25 for digestive system tumor) and hyperthyroidism (RR=3.00, 95%CI: 1.05-8.52 for HNSCC and RR=10.50, 95%CI: 4.54-24.26 for digestive system tumor). However, no significant increase was observed in the risk of hypophysitis, thyroiditis, diabetes mellitus, and adrenal insufficiency in these patient groups.

### 3.5 Subgroup analysis by different doses of PD-1 inhibitors

Guided by the dosage guidelines provided in the drug’s instructions and the Chinese Society of Clinical Oncology Immune Checkpoint Inhibitor Clinical Practice Guidelines, the studies were stratified into two groups for analysis: those receiving high-dose PD-1 inhibitors (≥ 10 mg/kg) and those receiving low-dose (< 10 mg/kg). We evaluated the risk of various immune-related adverse events across these dosage regimens. As illustrated in **Supplementary Figures S12**-**S14**, both dosage groups showed increased risks for hypothyroidism (RR=5.49, 95%CI: 4.25-7.09 for the low-dose group, and RR=19.08, 95%CI: 4.64-78.53 for the high-dose group) and hyperthyroidism (RR=10.33, 95%CI: 7.63-14.01 for the low-dose group, and RR=5.83, 95%CI: 1.91-17.85 for the high-dose group). A higher risk of hypophysitis was noted in the low-dose group (RR=4.95, 95%CI: 2.60-9.42), whereas this risk was not observed in patients receiving high-dose PD-1 inhibitors.

### 3.6 Subgroup analysis by previous treatment

Among the included randomized controlled trials, 39 studies involved patients who had previously received therapy for their malignancies, such as chemotherapy, radiotherapy, and biotherapy. In contrast, 10 studies focused on patients without previous treatments. Comparative analysis revealed that the risk of endocrine irAEs associated with PD-1 inhibitors was independent of whether patients had previously received treatments. Patients in the previously treated group had significantly increased risks for hypothyroidism (RR=5.85, 95%CI: 4.27-8.02), hyperthyroidism (RR=8.86, 95%CI: 6.26-12.55), hypophysitis (RR=3.97, 95%CI: 1.84-8.57), thyroiditis (RR=3.73, 95%CI: 1.84-7.56), diabetes mellitus (RR=2.23, 95%CI: 1.07-4.64), and adrenal insufficiency (RR=3.02, 95%CI: 1.52-6.01). Similarly, patients in the previously untreated group also exhibited significantly increased risks for hypothyroidism (RR=5.55, 95%CI: 3.50-8.79), hyperthyroidism (RR=13.10, 95%CI: 7.57-22.66), hypophysitis (RR=6.42, 95%CI: 2.25-18.33), thyroiditis (RR=6.72, 95%CI: 2.50-18.08), diabetes mellitus (RR=4.99, 95%CI: 1.45-17.17), and adrenal insufficiency (RR=7.81, 95%CI: 2.92-20.84) (**Supplementary Figures S15**-**S20**).

## 4 Discussion

To our knowledge, this systematic review and meta-analysis presented is the most thorough and detailed investigations into the risk of endocrine-related adverse effects in cancer patients treated with PD-1 inhibitors. We conducted subgroup analyses to discern the risks associated with different factors, including the types of PD-1 inhibitors, dosing regimens, previous treatments, specific endocrine disorders, and tumor types. Results demonstrated that the risks of endocrine dysfunctions including hypothyroidism, hyperthyroidism, hypophysitis, thyroiditis, diabetes mellitus, and adrenal insufficiency in patients treated solely with PD-1 inhibitors was significantly increased compared to those receiving chemotherapy, targeted therapies, or placebo treatments.

In this study, results indicated that the risk of thyroid dysfunctions significantly increased in the patient cohorts treated with PD-1 inhibitors. It is worth noting that the risks for hypothyroidism, hyperthyroidism, and thyroiditis varied, with hyperthyroidism posing the highest risk, followed by hypothyroidism, and then thyroiditis. Pembrolizumab and nivolumab were associated with an increased risk of hypothyroidism and hyperthyroidism. Additionally, pembrolizumab therapy was also associated with an increased risk of thyroiditis, while nivolumab did not exhibit a significant increase in the risk of thyroiditis. We found that tislelizumab and sintilimab significantly increased the risk of hypothyroidism, and this risk seemed to exceed that observed with pembrolizumab or nivolumab. It should be emphasized that this finding relies on a limited dataset from only two relevant studies. Therefore, further data from large clinical trials are needed to clarify this association. Regarding the endocrine dysfunctions associated with camrelizumab and toripalimab, each was reported in only one study, thus no relevant meta-analysis conclusions can be drawn currently. Subgroup analysis indicated that treatment with PD-1 inhibitors resulted in an increased risk for both hypothyroidism and hyperthyroidism, irrespective of the tumor types and the dosage of PD-1 inhibitors administered. Moreover, a significantly increased risk of thyroiditis was particularly observed in patients with NSCLC, whereas this increased risk was not significant in patients with melanoma or digestive system tumor. Furthermore, we found that the occurrence risk of endocrine-related adverse events was independent on previous cancer treatments. Thyroid toxicity has become a major endocrine adverse effect associated with ICPIs therapy, especially with PD-1 inhibitors. Our results were consistent with previous studies that PD-1 inhibitors were associated with an increased risk of hyperthyroidism and hypothyroidism (64). Although the specific pathways underlying this toxicity remain unclear, potential mechanisms may involve enhanced T-cell activation, stimulation of autoantibodies, and increased cytokine levels. PD-1 is expressed on mature CD4+ and CD8+ T cells, B cells, monocytes, as well as some subsets of dendritic cells, playing a crucial role in the inhibition of T-cell activation. PD-1 maintain the body’s immune homeostasis through the interaction with its two ligands, PD-L1 and PD-L2. Immune checkpoint inhibitors work by interfering with the PD-1 pathway, blocking PD-1 from binding to its ligands. This interruption releases self-reactive effector T cells that can destroy tumor cells (65). Since normal thyroid tissues express PD-1 ligands, they are notably vulnerable to attack by these cytotoxic T cells, resulting in thyroid impairment. Moreover, the excessive activation of effector T cells can trigger autoimmune responses in tissues, disrupting the balance of self-tolerance. This could trigger a series of inflammatory reactions, characterized by the release of cytokines such as interferon (IFN), tumor necrosis factor (TNF), and interleukin-2 (IL-2), further increasing the risk of thyroid dysfunction (66). Thyroid dysfunction caused by PD-1 inhibitors usually manifests within weeks to months after starting the medication. The initial clinical symptoms are subtle and non-specific. A large number of clinical observations have revealed that approximately half of the patients with impaired thyroid function were irreversible. If not promptly identified and intervened, this can directly impact patient’s prognosis. Hence, when employing PD-1 inhibitors in cancer patients, regular monitoring of thyroid function is needed. Patients with concurrent autoimmune diseases or a history of pre-existing thyroid disorders should receive closely monitoring of thyroid function upon starting PD-1 inhibitor therapy to ensure early diagnosis and timely intervention treatment.

In this study, PD-1 inhibitors were associated with an increased risk of hypophysitis. Subsequent subgroup analysis indicated that pembrolizumab increased the risk of hypophysitis, whereas this risk was not observed in patients treated with nivolumab monotherapy. This indicated that, as representative PD-1 inhibitors, pembrolizumab had a higher risk of hypophysitis compared to nivolumab, which was consistent with previous studies. Filette et al. (67) found in their 2019 study that the incidence of hypophysitis varied among PD-1 inhibitors, with a rate of 1.1% for pembrolizumab, which is higher than the 0.5% observed with nivolumab. This study did not include literature on hypophysitis induced by tislelizumab, sintilimab, camrelizumab, and toripalimab. Previous research has demonstrated that the occurrence of hypophysitis induced by ICPIs is not dose-dependent, with no significant difference in the incidence between the standard dose (3 mg/kg) and the cumulative or higher doses (10 mg/kg) of ICPIs (68, 69). Currently, there are few case reports on hypophysitis associated with PD-1 inhibitors, thus the incidence of hypophysitis induced by PD-1 inhibitors and its relationship to dosage require further investigation. Through subgroup analysis based on dosages, we found that cancer patients receiving low-dose treatment had an increased risk of hypophysitis. In contrast, those receiving high-dose PD-1 inhibitors did not exhibit an increased risk of hypophysitis, a finding that may be attributed to the limited inclusion of only three studies involving high-dose administration. Barroso-Sousa et al. (12) conducted a study on patients with various tumor types and found that those with melanoma had the highest risk of hypophysitis. In our study, patients with melanoma treated with PD-1 inhibitors showed a significantly increased risk of hypophysitis, whereas this risk was not observed in patients with NSCLC, HNSCC, and digestive system tumors. The pathogenesis of hypophysitis induced by PD-1 inhibitors is currently unclear. Researchers (70) found that patients with both IgG4-related hypophysitis and PD-1 inhibitor-induced hypophysitis had anti-pituitary antibodies or anti-pituitary hormone autoantibodies in their peripheral blood. This finding suggested that the pathogenesis of hypophysitis caused by PD-1 inhibitors might involve autoimmunity processes, similar to that of IgG4-related hypophysitis.

ICPIs-related adrenal insufficiency is a relatively uncommon endocrine adverse event. Previous research indicated that the incidence of adrenal insufficiency induced by PD-1 inhibitors varies between 0.38% and 0.87% (16, 17, 45). A study by Su et al. (71) found that the incidence of adrenal insufficiency caused by pembrolizumab was 0.67%. In this study, the use of PD-1 inhibitors was associated with an increased risk of adrenal insufficiency. Further subgroup analysis revealed that patients treated with pembrolizumab had a significantly increased risk of adrenal insufficiency. However, this risk was not observed in patients treated with nivolumab. Due to only one article being found on camrelizumab-induced adrenal insufficiency, the related pooled analysis was not conducted. Additionally, there are currently no literature on adrenal insufficiency caused by tislelizumab, sintilimab, and toripalimab monotherapy. Further subgroup analysis based on cancer types indicated that, patients with NSCLC who received PD-1 inhibitors monotherapy had a significantly increased risk of adrenal insufficiency. In contrast, this risk was not observed in patients with melanoma, HNSCC, and digestive system tumors. Previous studies have indicated that adrenal insufficiency associated with ICPIs is the result of drug-induced autoimmune adrenalitis, which may be co-mediated by a variety of mechanisms including autoreactive T lymphocytes, autoantibodies, and cytokines. Additionally, the polymorphisms of certain genes may also enhance the genetic susceptibility to autoimmune adrenalitis, including CTLA4, PD-1 genes, as well as the human leukocyte antigen haplotypes such as HLA-DR3-DQ2 and HLA-DR4-DQ8 (72). Due to the limited number of cases with primary adrenal insufficiency caused by ICPIs observed clinically, the exact pathogenic mechanisms and risk factors remain unclear.

Diabetes is a relatively uncommon endocrine immune-related adverse event associated with ICPIs, mainly observed in patients treated with PD-1 inhibitors. A retrospective analysis revealed that the incidence of ICPIs-related diabetes ranged from 0.9% to 1.0% (73). The World Health Organization’s safety report database showed a rising trend in diabetes cases associated with ICPIs (74). In this study, we found that PD-1 inhibitors significantly increased the risk of diabetes compared to the control groups. Further subgroup analysis showed that pembrolizumab significantly increased the risk of diabetes, while no increased risk was observed in nivolumab. A study by Su et al. (71) showed a diabetes incidence of 0.51% with pembrolizumab and 0.34% with nivolumab. However, according to a safety database from a Japanese pharmaceutical company (75), the incidence of diabetes were 0.33% with nivolumab and 0.14% with pembrolizumab. This study only included literature reporting diabetes associated with pembrolizumab, nivolumab, and camrelizumab. Since only one article reported diabetes induced by camrelizumab, no relevant pooled analysis was conducted. Subgroup analysis by tumor types showed that patients with melanoma had a significant increase in the risk of diabetes following PD-1 inhibitors therapy, whereas this risk was not observed among patients with NSCLC, HNSCC, and gastrointestinal tumors. ICPIs-related diabetes may be a novel form of autoimmune disease, possibly associated with the activation of autoreactive T lymphocytes and the generation of specific autoantigens. Studies have demonstrated that the interaction between PD-L1, expressed on islet cells, and PD-1 on activated T lymphocytes can suppress the tissue damage and cytokine release mediated by pathogenic autoreactive CD4+ T lymphocytes (76). Mouse model research has confirmed that inhibiting the PD1-PDL1 pathway can trigger autoimmune diabetes (77). By blocking the PD-1 pathway, PD-1 inhibitors can cause a decreased inhibition of cytotoxic T cells. This downregulation leads to the infiltration and destruction of pancreatic β-cells, ultimately triggering the onset of autoimmune diabetes. Therefore, in comparison to other ICPIs, PD-1 inhibitors are more prone to causing diabetes. Studies have demonstrated that the occurrence of autoimmune diabetes is associated with genetic factors, particularly in patients carrying high-risk HLA genotypes, such as HLA-DR4, which might heighten the susceptibility to diabetes (76). Nonetheless, the number of patients undergoing HLA typing is limited to date, and the exact role of genetic factors in the pathogenesis of ICPIs-related diabetes still requires deeper investigation.

A major strength of this study is that it exclusively includes research on PD-1 inhibitors, rather than all ICPIs, making the studies less heterogenous and results more reliable. Additionally, compared with previous reviews, this is the first meta-analysis to assess the risk of endocrine irAEs encompassing hypothyroidism, hyperthyroidism, hypophysitis, thyroiditis, diabetes mellitus, and adrenal insufficiency in patients treated with PD-1 inhibitors. Subgroup analyses were conducted as much as possible to make the results more convincing.

Our study has several limitations. First, the number of studies reporting thyroiditis, hypophysitis, adrenal insufficiency, and diabetes mellitus is relatively small, therefore some subgroup analyses were not conducted. Second, this meta-analysis utilized data from clinical trials with strict inclusion criteria, which may limit the applicability of our results to patients who do not meet the selection criteria for clinical trials. Thus, conducting large-scale real-world studies that includes a wide range of populations is necessary.

## 5 Conclusion

In conclusion, this study confirmed that PD-1 inhibitors significantly increased the risk of immune-induced endocrine disorders. Our findings indicated that pembrolizumab was associated with a higher risk of hypothyroidism, hyperthyroidism, thyroiditis, hypophysitis, diabetes mellitus, and adrenal insufficiency. Nivolumab was associated with a higher risk of hypothyroidism and hyperthyroidism. Both tislelizumab and sintilimab were associated with an increased risk of hypothyroidism. For different tumor types, patients treated with PD-1 inhibitors exhibited an increased risk of hypothyroidism and hyperthyroidism. Besides, patients with NSCLC had a higher risk of thyroiditis and adrenal insufficiency. Patients with melanoma were at a higher risk of hypophysitis and diabetes mellitus. Both low- and high-dose group of PD-1 inhibitors were at higher risk of hypothyroidism and hyperthyroidism. Enhanced awareness and understanding of the endocrinopathy risk profiles associated with PD-1 inhibitors are crucial for clinicians.

## Data availability statement

The original contributions presented in the study are included in the article/**Supplementary Material**. Further inquiries can be directed to the corresponding author.

## Author contributions

PZ: Data curation, Methodology, Software, Project administration, Writing – original draft, Writing – review & editing. TZ: Data curation, Conceptualization, Investigation, Writing – review & editing. LY: Writing – review & editing, Formal analysis, Methodology, Software, Validation. WM: Methodology, Software, Writing – review & editing. WL: Methodology, Software, Data curation, Writing – review & editing. CZ: Project administration, Writing – original draft, Writing – review & editing, Data curation, Funding acquisition, Methodology, Validation.

## References

[1] Albarran-Artahona V Laguna JC Gorria T Torres-Jimenez J Pascal M Mezquita L . Immune-related uncommon adverse events in patients with cancer treated with immunotherapy. Diagnostics (Basel). (2022) 12:1–31. doi: 10.3390/diagnostics12092091 PMC9498261 36140493

[2] Albiges L Tannir NM Burotto M McDermott D Plimack ER Barthélémy P . Nivolumab plus ipilimumab versus sunitinib for first-line treatment of advanced renal cell carcinoma: extended 4-year follow-up of the phase III CheckMate 214 trial. ESMO Open. (2020) 5:e001079. doi: 10.1136/esmoopen-2020-001079 33246931 PMC7703447

[3] Paz-Ares L Ciuleanu TE Cobo M Schenker M Zurawski B Menezes J . First-line nivolumab plus ipilimumab combined with two cycles of chemotherapy in patients with non-small-cell lung cancer (CheckMate 9LA): an international, randomised, open-label, phase 3 trial. Lancet Oncol. (2021) 22:198–211. doi: 10.1016/S1470-2045(20)30641-0 33476593

[4] Cramer P Bresalier RS . Gastrointestinal and hepatic complications of immune checkpoint inhibitors. Curr Gastroenterol Rep. (2017) 19:3. doi: 10.1007/s11894-017-0540-6 28124291

[5] Hofmann L Forschner A Loquai C Goldinger SM Zimmer L Ugurel S . Cutaneous, gastrointestinal, hepatic, endocrine, and renal side-effects of antiPD-1 therapy. Eur J Cancer. (2016) 60:190–209. doi: 10.1016/j.ejca.2016.02.025 27085692

[6] Wang Y Zhou S Yang F Qi X Wang X Guan X . Treatment-related adverse events of PD-1 and PD-L1 inhibitors in clinical trials: a systematic review and meta-analysis. JAMA Oncol. (2019) 5:1008–19. doi: 10.1001/jamaoncol.2019.0393 PMC6487913 31021376

[7] Wang DY Salem JE Cohen JV Chandra S Menzer C Ye F . Fatal toxic effects associated with immune checkpoint inhibitors: a systematic review and Meta analysis. JAMA Oncol. (2018) 4:1721–8. doi: 10.1001/jamaoncol.2018.3923 PMC6440712 30242316

[8] Villadolid J Amin A . Immune checkpoint inhibitors in clinical practice: update on management of immune-related toxicities. Transl Lung Cancer Res. (2015) 4:560–75. doi: 10.3978/j.issn.2218-6751.2015.06.06 PMC4630514 26629425

[9] Spain L Diem S Larkin J . Management of toxicities of immune checkpoint inhibitors. Cancer Treat Rev. (2016) 44):51–60. doi: 10.1016/j.ctrv.2016.02.001 26874776

[10] Wolchok JD Chiarion-Sileni V Gonzalez R Rutkowski P Grob JJ Cowey CL . Overall survival with combined nivolumab and ipilimumab in advanced melanoma. N Engl J Med. (2017) 377:1345–56. doi: 10.1056/NEJMoa1709684 PMC5706778 28889792

[11] Martin-Liberal J Furness AJ Joshi K Peggs KS Quezada SA Larkin J . Anti-programmed cell death-1 therapy and insulin-dependent diabetes: a case report. Cancer Immunol Immunother. (2015) 64:765–7. doi: 10.1007/s00262-015-1689-1 PMC11028478 25828465

[12] Barroso-Sousa R Barry WT Garrido-Castro AC Hodi FS Min L Krop IE . Incidence of endocrine dysfunction following the use of different immune checkpoint inhibitor regimens: A systematic review and meta-analysis. JAMA Oncol. (2018) 4:173–82. doi: 10.1001/jamaoncol.2017.3064 PMC5838579 28973656

[13] Duan L Wang L Si X Zhang L Liu X Li Y . Clinical diagnosis and treatment of immune-related adverse events of edocrine system related to immune checkpoint inhibitors. Zhongguo Fei Ai Za Zhi. (2019) 22:649–52. doi: 10.3779/j.issn.1009-3419.2019.10.08 PMC6817430 31650948

[14] Higgins JPT Altman DG JACS . Chapter 8: Assessing risk of bias in included studies. In: Higgins JPT Churchill R Chandler J MS C . editors. Cochrane handbook for systematic reviews of interventions. version 5.2.0. Cochrane (2017). Available at: https://training.cochrane.org/handbook.

[15] Page MJ McKenzie JE Bossuyt PM Boutron I Hoffmann TC Mulrow CD . The PRISMA 2020 statement: An updated guideline for reporting systematic reviews. BMJ. (2021) 372:n71. doi: 10.1136/bmj.n71 33782057 PMC8005924

[16] Herbst RS Baas P Kim DW Felip E Pérez-Gracia JL Han JY . Pembrolizumab versus docetaxel for previously treated, PD-L1-positive, advanced non-small-cell lung cancer (KEYNOTE-010): a randomised controlled trial. Lancet. (2016) 387:1540–50. doi: 10.1016/S0140-6736(15)01281-7 26712084

[17] Bellmunt J de Wit R Vaughn DJ Fradet Y Lee JL Fong L . Pembrolizumab as second-line therapy for advanced urothelial carcinoma. N Engl J Med. (2017) 376:1015–26. doi: 10.1056/NEJMoa1613683 PMC5635424 28212060

[18] Cohen EEW Soulières D Le Tourneau C Dinis J Licitra L Ahn MJ . Pembrolizumab versus methotrexate, docetaxel, or cetuximab for recurrent or metastatic head-and-neck squamous cell carcinoma (KEYNOTE-040): a randomised, open-label, phase 3 study. Lancet. (2019) 393:156–67. doi: 10.1016/S0140-6736(18)31999-8 30509740

[19] Eggermont AMM Blank CU Mandala M Long GV Atkinson V Dalle S . Adjuvant pembrolizumab versus placebo in resected stage III melanoma. N Engl J Med. (2018) 378:1789–801. doi: 10.1056/NEJMoa1802357 29658430

[20] Finn RS Ryoo BY Merle P Kudo M Bouattour M Lim HY . Pembrolizumab as second-line therapy in patients with advanced hepatocellular carcinoma in KEYNOTE-240: A randomized, double-blind, phase III trial. J Clin Oncol. (2020) 38:193–202. doi: 10.1200/JCO.19.01307 31790344

[21] Shitara K Özgüroğlu M Bang YJ Di Bartolomeo M Mandalà M Ryu MH . Pembrolizumab versus paclitaxel for previously treated, advanced gastric or gastro-oesophageal junction cancer (KEYNOTE-061): a randomised, open-label, controlled, phase 3 trial. Lancet. (2018) 392:123–33. doi: 10.1016/S0140-6736(18)31257-1 29880231

[22] Kuruvilla J Ramchandren R Santoro A Paszkiewicz-Kozik E Gasiorowski R Johnson NA . Pembrolizumab versus brentuximab vedotin in relapsed or refractory classical Hodgkin lymphoma (KEYNOTE-204): an interim analysis of a multicentre, randomised, open-label, phase 3 study. Lancet Oncol. (2021) 22:512–24. doi: 10.1016/S1470-2045(21)00005-X 33721562

[23] Shitara K Van Cutsem E Bang YJ Fuchs C Wyrwicz L Lee KW . Efficacy and safety of pembrolizumab or pembrolizumab plus chemotherapy vs chemotherapy alone for patients with first-line, advanced gastric cancer: the KEYNOTE-062 phase 3 randomized clinical trial. JAMA Oncol. (2020) 6:1571–80. doi: 10.1001/jamaoncol.2020.3370 PMC7489405 32880601

[24] Winer EP Lipatov O Im SA Goncalves A Muñoz-Couselo E Lee KS . Pembrolizumab versus investigator-choice chemotherapy for metastatic triple-negative breast cancer (KEYNOTE-119): a randomised, open-label, phase 3 trial. Lancet Oncol. (2021) 22:499–511. doi: 10.1016/S1470-2045(20)30754-3 33676601

[25] Powles T Csőszi T Özgüroğlu M Matsubara N Géczi L Cheng SY . Pembrolizumab alone or combined with chemotherapy versus chemotherapy as first-line therapy for advanced urothelial carcinoma (KEYNOTE-361): a randomised, open-label, phase 3 trial. Lancet Oncol. (2021) 22:931–45. doi: 10.1016/S1470-2045(21)00152-2 34051178

[26] André T Shiu KK Kim TW Jensen BV Jensen LH Punt C . Pembrolizumab in microsatellite-instability-high advanced colorectal cancer. N Engl J Med. (2020) 383:2207–18. doi: 10.1056/NEJMoa2017699 33264544

[27] Kojima T Shah MA Muro K Francois E Adenis A Hsu CH . Randomized phase III KEYNOTE-181 study of pembrolizumab versus chemotherapy in advanced esophageal cancer. J Clin Oncol. (2020) 38:4138–48. doi: 10.1200/JCO.20.01888 33026938

[28] Burtness B Harrington KJ Greil R Soulières D Tahara M de Castro G Jr . Pembrolizumab alone or with chemotherapy versus cetuximab with chemotherapy for recurrent or metastatic squamous cell carcinoma of the head and neck (KEYNOTE-048): a randomised, open-label, phase 3 study. Lancet. (2019) 394:1915–28. doi: 10.1016/S0140-6736(19)32591-7 31679945

[29] Galsky MD Mortazavi A Milowsky MI George S Gupta S Fleming MT . Randomized double-blind phase II study of maintenance pembrolizumab versus placebo after first-line chemotherapy in patients with metastatic urothelial cancer. J Clin Oncol. (2020) 38:1797–806. doi: 10.1200/JCO.19.03091 PMC7255983 32271672

[30] Wakelee H Liberman M Kato T Tsuboi M Lee SH Gao S . Perioperative pembrolizumab for early-stage non-small-cell lung cancer. N Engl J Med. (2023) 389:491–503. doi: 10.1056/NEJMoa2302983 37272513 PMC11074923

[31] Qin S Chen Z Fang W Ren Z Xu R Ryoo BY . Pembrolizumab versus placebo as second-line therapy in patients from Asia with advanced hepatocellular carcinoma: A randomized, double-blind, phase III trial. J Clin Oncol. (2023) 41:1434–43. doi: 10.1200/JCO.22.00620 PMC9995104 36455168

[32] Powles T Tomczak P Park SH Venugopal B Ferguson T Symeonides SN . Pembrolizumab versus placebo as post-nephrectomy adjuvant therapy for clear cell renal cell carcinoma (KEYNOTE-564): 30-month follow-up analysis of a multicentre, randomised, double-blind, placebo-controlled, phase 3 trial. Lancet Oncol. (2022) 23:1133–44. doi: 10.1016/S1470-2045(22)00487-9 36055304

[33] Long GV Luke JJ Khattak MA de la Cruz Merino L Del Vecchio M Rutkowski P . Pembrolizumab versus placebo as adjuvant therapy in resected stage IIB or IIC melanoma (KEYNOTE-716): distant metastasis-free survival results of a multicentre, double-blind, randomised, phase 3 trial. Lancet Oncol. (2022) 23:1378–88. doi: 10.1016/S1470-2045(22)00559-9 36265502

[34] Chung HC Kang YK Chen Z Bai Y Wan Ishak WZ Shim BY . Pembrolizumab versus paclitaxel for previously treated advanced gastric or gastroesophageal junction cancer (KEYNOTE-063): A randomized, open-label, phase 3 trial in Asian patients. Cancer. (2022) 128:995–1003. doi: 10.1002/cncr.34019 34878659 PMC9299889

[35] Chan ATC Lee VHF Hong RL Ahn MJ Chong WQ Kim SB . Pembrolizumab monotherapy versus chemotherapy in platinum-pretreated, recurrent or metastatic nasopharyngeal cancer (KEYNOTE-122): an open-label, randomized, phase III trial. Ann Oncol. (2023) 34:251–61. doi: 10.1016/j.annonc.2022.12.007 36535566

[36] Popat S Curioni-Fontecedro A Dafni U Shah R O'Brien M Pope A . A multicentre randomised phase III trial comparing pembrolizumab versus single-agent chemotherapy for advanced pre-treated Malignant pleural mesothelioma: the European Thoracic Oncology Platform (ETOP 9-15) PROMISE-meso trial. Ann Oncol. (2020) 31:1734–45. doi: 10.1016/j.annonc.2020.09.009 32976938

[37] Ren S Feng J Ma S Chen H Ma Z Huang C . KEYNOTE-033: Randomized phase 3 study of pembrolizumab vs docetaxel in previously treated, PD-L1-positive, advanced NSCLC. Int J Cancer. (2023) 153:623–34. doi: 10.1002/ijc.34532 37141294

[38] Reck M Rodríguez-Abreu D Robinson AG Hui R Csőszi T Fülöp A . Five-year outcomes with pembrolizumab versus chemotherapy for metastatic non-small-cell lung cancer with PD-L1 tumor proportion score ≥ 50%. J Clin Oncol. (2021) 39:2339–49. doi: 10.1200/JCO.21.00174 PMC8280089 33872070

[39] de Castro G Jr Kudaba I Wu YL Lopes G Kowalski DM Turna HZ . Five-year outcomes with pembrolizumab versus chemotherapy as first-line therapy in patients with non-small-cell lung cancer and programmed death ligand-1 tumor proportion score ≥ 1% in the KEYNOTE-042 study. J Clin Oncol. (2023) 41:1986–91. doi: 10.1200/JCO.21.02885 PMC10082298 36306479

[40] Hamid O Puzanov I Dummer R Schachter J Daud A SChadendorf D . Final analysis of a randomised trial comparing pembrolizumab versus investigator-choice chemotherapy for ipilimumab-refractory advanced melanoma. Eur J Cancer. (2017) 86:37–45. doi: 10.1016/j.ejca.2017.07.022 28961465

[41] Pelosof L Saung MT Donoghue M Casak S Mushti S Cheng J . Benefit-risk summary of nivolumab for the treatment of patients with unresectable advanced, recurrent, or metastatic esophageal squamous cell carcinoma after prior fluoropyrimidine- and platinum-based chemotherapy. Oncologist. (2021) 26:318–24. doi: 10.1002/onco.13646 PMC8018317 33345396

[42] Kelly RJ Ajani JA Kuzdzal J Zander T Van Cutsem E Piessen G . Adjuvant nivolumab in resected esophageal or gastroesophageal junction cancer. N Engl J Med. (2021) 384:1191–203. doi: 10.1056/NEJMoa2032125 33789008

[43] Wu YL Lu S Cheng Y Zhou C Wang J Mok T . Nivolumab versus docetaxel in a predominantly Chinese patient population with previously treated advanced NSCLC: checkMate 078 randomized phase III clinical trial. J Thorac Oncol. (2019) 14:867–75. doi: 10.1016/j.jtho.2019.01.006 30659987

[44] Hellmann MD Ciuleanu TE Pluzanski A Lee JS Otterson GA Audigier-Valette C . Nivolumab plus ipilimumab in lung cancer with a high tumor mutational burden. N Engl J Med. (2018) 378:2093–104. doi: 10.1056/NEJMoa1801946 PMC7193684 29658845

[45] Ferris RL Blumenschein G Jr Fayette J Guigay J Colevas AD Licitra L . Nivolumab for recurrent squamous-cell carcinoma of the head and neck. N Engl J Med. (2016) 375:1856–67. doi: 10.1056/NEJMoa1602252 PMC5564292 27718784

[46] Robert C Long GV Brady B Dutriaux C Maio M Mortier L . Nivolumab in previously untreated melanoma without BRAF mutation. N Engl J Med. (2015) 372:320–30. doi: 10.1056/NEJMoa1412082 25399552

[47] Brahmer J Reckamp KL Baas P Crinò L Eberhardt WE Poddubskaya E . Nivolumab versus docetaxel in advanced squamous-cell non-small-cell lung cancer. N Engl J Med. (2015) 373:123–35. doi: 10.1056/NEJMoa1504627 PMC4681400 26028407

[48] Borghaei H Paz-Ares L Horn L Spigel DR Steins M Ready NE . Nivolumab versus docetaxel in advanced nonsquamous non-small-cell lung cancer. N Engl J Med. (2015) 373:1627–39. doi: 10.1056/NEJMoa1507643 PMC5705936 26412456

[49] Kang YK Boku N Satoh T Ryu MH Chao Y Kato K . Nivolumab in patients with advanced gastric or gastro-oesophageal junction cancer refractory to, or intolerant of, at least two previous chemotherapy regimens (ONO-4538-12, ATTRACTION-2): a randomised, double-blind, placebo-controlled, phase 3 trial. Lancet. (2017) 390:2461–71. doi: 10.1016/S0140-6736(17)31827-5 28993052

[50] Carbone DP Reck M Paz-Ares L Creelan B Horn L Steins M . First-line nivolumab in stage IV or recurrent non-small-cell lung cancer. N Engl J Med. (2017) 376:2415–26. doi: 10.1056/NEJMoa1613493 PMC6487310 28636851

[51] Zimmer L Livingstone E Hassel JC Fluck M Eigentler T Loquai C . Adjuvant nivolumab plus ipilimumab or nivolumab monotherapy versus placebo in patients with resected stage IV melanoma with no evidence of disease (IMMUNED): a randomised, double-blind, placebo-controlled, phase 2 trial. Lancet. (2020) 395:1558–68. doi: 10.1016/S0140-6736(20)30417-7 32416781

[52] Larkin J Minor D D'Angelo S Neyns B Smylie M Miller WH Jr . Overall survival in patients with advanced melanoma who received nivolumab versus investigator's choice chemotherapy in checkMate 037: A randomized, controlled, open-label phase III trial. J Clin Oncol. (2018) 36:383–90. doi: 10.1200/JCO.2016.71.8023 PMC6804912 28671856

[53] Fennell DA Ewings S Ottensmeier C Califano R GG H Hill K . Nivolumab versus placebo in patients with relapsed Malignant mesothelioma (CONFIRM): a multicentre, double-blind, randomised, phase 3 trial. Lancet Oncol. (2021) 22:1530–40. doi: 10.1016/S1470-2045(21)00471-X PMC8560642 34656227

[54] Hamanishi J Takeshima N Katsumata N Ushijima K Kimura T Takeuchi S . Nivolumab versus gemcitabine or pegylated liposomal doxorubicin for patients with platinum-resistant ovarian cancer: open-label, randomized trial in Japan (NINJA). J Clin Oncol. (2021) 39:3671–81. doi: 10.1200/JCO.21.00334 PMC8601279 34473544

[55] Reardon DA Brandes AA Omuro A Mulholland P Lim M Wick A . Effect of nivolumab vs bevacizumab in patients with recurrent glioblastoma: the checkMate 143 phase 3 randomized clinical trial. JAMA Oncol. (2020) 6:1003–10. doi: 10.1001/jamaoncol.2020.1024 PMC7243167 32437507

[56] Bajorin DF Witjes JA Gschwend JE Schenker M Valderrama BP Tomita Y . Adjuvant nivolumab versus placebo in muscle-invasive urothelial carcinoma. N Engl J Med. (2021) 384:2102–14. doi: 10.1056/NEJMoa2034442 PMC8215888 34077643

[57] Yau T Park JW Finn RS Cheng AL Mathurin P Edeline J . Nivolumab versus sorafenib in advanced hepatocellular carcinoma (CheckMate 459): a randomised, multicentre, open-label, phase 3 trial. Lancet Oncol. (2022) 23:77–90. doi: 10.1016/S1470-2045(21)00604-5 34914889

[58] Shen L Kato K Kim SB Ajani JA Zhao K He Z . Tislelizumab versus chemotherapy as second-line treatment for advanced or metastatic esophageal squamous cell carcinoma (RATIONALE-302): A randomized phase III study. J Clin Oncol. (2022) 40:3065–76. doi: 10.1200/JCO.21.01926 PMC9462531 35442766

[59] Zhou C Huang D Fan Y Yu X Liu Y Shu Y . Tislelizumab versus docetaxel in patients with previously treated advanced NSCLC (RATIONALE-303): A phase 3, open-label, randomized controlled trial. J Thorac Oncol. (2023) 18:93–105. doi: 10.1016/j.jtho.2022.09.217 36184068

[60] Xu J Li Y Fan Q Shu Y Yang L Cui T . Clinical and biomarker analyses of sintilimab versus chemotherapy as second-line therapy for advanced or metastatic esophageal squamous cell carcinoma: a randomized, open-label phase 2 study (ORIENT-2). Nat Commun. (2022) 13:857. doi: 10.1038/s41467-022-28408-3 35165274 PMC8844279

[61] Shi Y Wu L Yu X Xing P Wang Y Zhou J . Sintilimab versus docetaxel as second-line treatment in advanced or metastatic squamous non-small-cell lung cancer: an open-label, randomized controlled phase 3 trial (ORIENT-3). Cancer Commun (Lond). (2022) 42:1314–30. doi: 10.1002/cac2.12385 PMC9759762 36336841

[62] Huang J Xu J Chen Y Zhuang W Zhang Y Chen Z . Camrelizumab versus investigator's choice of chemotherapy as second-line therapy for advanced or metastatic oesophageal squamous cell carcinoma (ESCORT): a multicentre, randomised, open-label, phase 3 study. Lancet Oncol. (2020) 21:832–42. doi: 10.1016/S1470-2045(20)30110-8 32416073

[63] Lian B Si L Chi ZH Sheng XN Kong Y Wang X . Toripalimab (anti-PD-1) versus high-dose interferon-α2b as adjuvant therapy in resected mucosal melanoma: a phase II randomized trial. Ann Oncol. (2022) 33:1061–70. doi: 10.1016/j.annonc.2022.07.002 35842199

[64] Trevisani V Iughetti L Lucaccioni L Predieri B . Endocrine immune-related adverse effects of immune-checkpoint inhibitors. Expert Rev Endocrinol Metab. (2023) 18:441–51. doi: 10.1080/17446651.2023.2256841 37682107

[65] Cardona Z Sosman JA Chandra S Huang W . Endocrine side effects of immune checkpoint inhibitors. Front Endocrinol (Lausanne). (2023) 14:1157805. doi: 10.3389/fendo.2023.1157805 37251665 PMC10210589

[66] Wright JJ Powers AC Johnson DB . Endocrine toxicities of immune checkpoint inhibitors. Nat Rev Endocrinol. (2021) 17:389–99. doi: 10.1038/s41574-021-00484-3 PMC8769055 33875857

[67] de Filette J Andreescu CE Cools F Bravenboer B Velkeniers B . A systematic review and meta-analysis of endocrine-related adverse events associated with immune checkpoint inhibitors. Horm Metab Res. (2019) 51:145–56. doi: 10.1055/a-0843-3366 30861560

[68] Faje A . Immunotherapy and hypophysitis: clinical presentation, treatment, and biologic insights. Pituitary. (2016) 19:82–92. doi: 10.1007/s11102-015-0671-4 26186958

[69] Faje AT Sullivan R Lawrence D Tritos NA Fadden R Klibanski A . Ipilimumab-induced hypophysitis: a detailed longitudinal analysis in a large cohort of patients with metastatic melanoma. J Clin Endocrinol Metab. (2014) 99:4078–85. doi: 10.1210/jc.2014-2306 25078147

[70] Kanie K Iguchi G Bando H Urai S Shichi H Fujita Y . Mechanistic insights into immune checkpoint inhibitor-related hypophysitis: a form of paraneoplastic syndrome. Cancer Immunol Immunother. (2021) 70:3669–77. doi: 10.1007/s00262-021-02955-y PMC8571153 33977343

[71] Su Q Zhang XC Wang DY Zhang HR Zhu C Hou YL . The risk of immune-related endocrine disorders associated with anti-PD-1 inhibitors therapy for solid tumors: A systematic review and meta-analysis. Int Immunopharmacol. (2018) 59:328–38. doi: 10.1016/j.intimp.2018.04.021 29679857

[72] Betterle C Morlin L . Autoimmune Addison's disease. Endocr Dev. (2011) 20:161–72. doi: 10.1159/000321239 21164269

[73] Stamatouli AM Quandt Z Perdigoto AL Clark PL Kluger H Weiss SA . Collateral damage: insulin-dependent diabetes induced with checkpoint inhibitors. Diabetes. (2018) 67:1471–80. doi: 10.2337/dbi18-0002 PMC6054443 29937434

[74] Wright JJ Salem JE Johnson DB Lebrun-Vignes B Stamatouli A Thomas JW . Increased reporting of immune checkpoint inhibitor-associated diabetes. Diabetes Care. (2018) 41:e150–1. doi: 10.2337/dc18-1465 PMC7301161 30305348

[75] Baden MY Imagawa A Abiru N Awata T Ikegami H Uchigata Y . Characteristics and clinical course of type 1 diabetes mellitus related to anti-programmed cell death-1 therapy. Diabetol Int. (2018) 10:58–66. doi: 10.1007/s13340-018-0362-2 30800564 PMC6357237

[76] Quandt Z Young A Anderson M . Immune checkpoint inhibitor diabetes mellitus: a novel form of autoimmune diabetes. Clin Exp Immunol. (2020) 200:131–40. doi: 10.1111/cei.13424 PMC7160652 32027018

[77] Ansari MJ Salama AD Chitnis T Smith RN Yagita H Akiba H . The programmed death-1 (PD-1) pathway regulates autoimmune diabetes in nonobese diabetic (NOD) mice. J Exp Med. (2003) 198:63–9. doi: 10.1084/jem.20022125 PMC2196083 12847137

