# Supplementary material for: The risk of endocrine immune-related adverse events induced by PD-1 inhibitors in cancer patients: a systematic review and meta-analysis
Source: Front Oncol. 2024 May 2;14:1381250. doi: 10.3389/fonc.2024.1381250 (PMC11096456; doi:10.3389/fonc.2024.1381250)
Supplement: Supplementary file 1 [file DataSheet_1.docx]

**Supplementary Material**

**Figure Captions**

**Figure S1.** Risk of bias item presented as percentages across all included studies.

**Figure S2.** Risk of bias summary and graph.

**Figure S3.** Forest plots of the relative risks of hypophysitis related to different PD-1 inhibitors.

**Figure S4.** Forest plots of the relative risks of adrenal insufficiency related to different PD-1 inhibitors.

**Figure S5.** Forest plots of the relative risks of diabetes mellitus related to different PD-1 inhibitors.

**Figure S6.** Forest plots of the relative risks of hypothyroidism related to different tumor types.

**Figure S7.** Forest plots of the relative risks of hyperthyroidism related to different tumor types.

**Figure S8.** Forest plots of the relative risks of thyroiditis related to different tumor types.

**Figure S9.** Forest plots of the relative risks of hypophysitis related to different tumor types.

**Figure S10.** Forest plots of the relative risks of adrenal insufficiency related to different tumor types.

**Figure** **S11.** Forest plots of the relative risks of diabetes mellitus related to different tumor types.

**Figure S12.** Forest plots of the relative risks of hypothyroidism related to different doses of PD-1 inhibitors.

**Figure S13.** Forest plots of the relative risks of hyperthyroidism related to different doses of PD-1 inhibitors.

**Figure S14.** Forest plots of the relative risks of hypophysitis related to different doses of PD-1 inhibitors.

**Figure S15.** Forest plots of the relative risks of hypothyroidism related to previous treatments.

**Figure S16.** Forest plots of the relative risks of hyperthyroidism related to previous treatments.

**Figure S17.** Forest plots of the relative risks of thyroiditis related to previous treatments.

**Figure S18.** Forest plots of the relative risks of hypophysitis related to previous treatments.

**Figure S19.** Forest plots of the relative risks of adrenal insufficiency related to previous treatments.

**Figure S20.** Forest plots of the relative risks of diabetes mellitus related to previous treatments.

**Table Captions**

**Table S1**  Literature search strategy.

**Table S2** Characteristics of the included studies for hypothyroidism.

**Table S3** Characteristics of the included studies for hyperthyroidism.

**Table S4** Characteristics of the included studies for thyroiditis.

**Table S5** Characteristics of the included studies for hypophysitis.

**Table S6** Characteristics of the included studies for adrenal insufficiency.

**Table S7** Characteristics of the included studies for diabetes mellitus.

**Table S1** **Literature search strategy.**

**Pubmed**

| Searches | | Results |
| --- | --- | --- |
| ^#^1 | pembrolizumab[Title/Abstract] OR nivolumab[Title/Abstract] OR camrelizumab[Title/Abstract] OR toripalimab[Title/Abstract] OR tislelizumab[Title/Abstract] OR sintilimab[Title/Abstract] | 15353 |
| ^#^2 | randomized controlled trial[Publication Type] OR randomized[Title/Abstract] OR placebo[Title/Abstract] | 1021350 |
| ^#^3 | ^#^1 AND ^#^2 | 1460 |

**Embase**

| Searches | | Results |
| --- | --- | --- |
| ^#^1 | 'pembrolizumab':ab,ti or 'nivolumab':ab,ti or 'camrelizumab':ab,ti or 'toripalimab':ab,ti or 'tislelizumab':ab,ti or 'sintilimab':ab,ti | 33145 |
| ^#^2 | 'randomized controlled trial':ab,ti or 'randomized':ab,ti or 'placebo':ab,ti | 1137472 |
| ^#^3 | ^#^1 AND ^#^2 | 4448 |

**the Cochrane Library**

| Searches | | Results |
| --- | --- | --- |
| ^#^1 | (pembrolizumab):ti,ab,kw or (nivolumab):ti,ab,kw or (camrelizumab):ti,ab,kw or (toripalimab):ti,ab,kw or (tislelizumab):ti,ab,kw or (sintilimab):ti,ab,kw | 5986 |
| ^#^2 | (randomized controlled trial):ti,ab,kw or (randomized):ti,ab,kw or (placebo):ti,ab,kw | 1358207 |
| ^#^3 | #1 and #2 | 4593 |

**Table S2 Characteristics of the included studies for hypothyroidism.**

| **Author / year** | **RCT number** | **Tumor types** | **Previous treatment** | **Intervention** | **Control** | **Age (year, I/C)** | **Gender (%male, I/C)** | **Sample size (I/C)** |
| --- | --- | --- | --- | --- | --- | --- | --- | --- |
|  |  |  |  |  |  |  |  |  |
| Herbst 1 2016 | NCT01905657 | NSCLC | Yes | Pembrolizumab 2 mg/kg Q3W | Chemotherapy | 63/62 | 62/61 | 648 (339/309) |
| Herbst 2 2016 | NCT01905657 | NSCLC | Yes | Pembrolizumab 10 mg/kg Q3W | Chemotherapy | 63/62 | 62/61 | 652 (343/309) |
| Bellmunt 2017 | NCT02256436 | Urothelial carcinoma | Yes | Pembrolizumab 200 mg Q3W | Chemotherapy | 67/65 | 74.1/74.3 | 521 (266/255) |
| Cohen 2019 | NCT02252042 | Head-and-neck squamous cell carcinoma | Yes | Pembrolizumab 200 mg Q3W | Chemotherapy | 60/60 | 84/83 | 480 (246/234) |
| Eggermont 2018 | NCT02362594 | Melanoma | Yes | Pembrolizumab 200 mg Q3W | Placebo | 54/54 | 63/60.2 | 1011 (509/502) |
| Finn 2020 | NCT02702401 | Hepatocellular Carcinoma | Yes | Pembrolizumab 200 mg Q3W | Placebo | 67/65 | 81.3/83 | 413 (279/134) |
| Shitara 2018 | NCT02370498 | Gastric or gastrooesophageal junction cancer | Yes | Pembrolizumab 200 mg Q3W | Chemotherapy | 62.5/60 | 68/70 | 570 (294/276) |
| Kuruvilla 2021 | NCT02684292 | Hodgkin lymphoma | Yes | Pembrolizumab 200 mg Q3W | Targeted therapy | 36/35 | 56/59 | 300 (148/152) |
| Shitara 2020 | NCT02494583 | Gastric cancer | No | Pembrolizumab 200 mg Q3W | Chemotherapy | 61/62.5 | 70.3/71.6 | 498 (254/244) |
| Winer 2021 | NCT02555657 | Metastatic triple-negative breast cancer | Yes | Pembrolizumab 200 mg Q3W | Chemotherapy | 50/53 | 0/1 | 601 (309/292) |
| Powles 2021 | NCT02853305 | Urothelial carcinoma | No | Pembrolizumab 200 mg Q3W | Chemotherapy | 68/69 | 74/74 | 644 (302/342) |
| André 2020 | NCT02563002 | Colorectal cancer | Yes | Pembrolizumab 200 mg Q3W | Chemotherapy | 63/62.5 | 46/53 | 296 (153/143) |
| Kojima 2020 | NCT02564263 | Esophageal cancer | Yes | Pembrolizumab 200 mg Q3W | Chemotherapy | 63/62 | 86.9/86.3 | 610 (314/296) |
| Burtness 2019 | NCT02358031 | Head-and-neck squamous cell carcinoma | No | Pembrolizumab 200 mg Q3W | Chemotherapy | 62/61 | 83/87 | 587 (300/287) |
| Galsky 2020 | NCT02500121 | Urothelial carcinoma | Yes | Pembrolizumab 200 mg Q3W | Placebo | 65/68 | 81/71 | 107 (55/52) |
| Wakelee 2023 | NCT03425643 | NSCLC | Yes | Pembrolizumab 200 mg Q3W | Placebo | 63/64 | 70.3/71 | 557 (290/267) |
| Qin 2023 | NCT03062358 | Hepatocellular carcinoma | Yes | Pembrolizumab 200 mg Q3W | Placebo | 54/54 | 85.7/82.4 | 452 (299/153) |
| Powles 2022 | NCT03142334 | Clear cell renal cell carcinoma | No | Pembrolizumab 200 mg Q3W | Placebo | 60/60 | 70/72 | 984 (488/496) |
| Long 2022 | NCT03553836 | Melanoma | No | Pembrolizumab 200 mg Q3W | Placebo | 60/61 | 62/59 | 969 (483/486) |
| Chung 2022 | NCT03019588 | Gastric or gastroesophageal junction cancer | Yes | Pembrolizumab 200 mg Q3W | Chemotherapy | 61/61 | 68/79 | 91 (47/44) |
| Chan 2023 | NCT02611960 | Nasopharyngeal cancer | Yes | Pembrolizumab 200 mg Q3W | Chemotherapy | 51/53 | 83.8/81.9 | 228 (116/112) |
| Popat 2020 | NCT02991482 | Malignant pleural mesothelioma | Yes | Pembrolizumab 200 mg Q3W | Chemotherapy | 70/69 | 81.9/79.4 | 142 (72/70) |
| Ren 2023 | NCT02864394 | NSCLC | Yes | Pembrolizumab 2 mg/kg Q3W | Chemotherapy | 61/61 | 73.7/77.4 | 411 (213/198) |
| Reck 2021 | NCT02142738 | NSCLC | No | Pembrolizumab 200 mg Q3W | Chemotherapy | 64.5/66 | 59.7/62.9 | 304 (154/150) |
| Castro 2023 | NCT02220894 | NSCLC | No | Pembrolizumab 200 mg Q3W | Chemotherapy | 63/63 | 71/71 | 1251 (636/615) |
| Hamid 1 2017 | NCT01704287 | Melanoma | Yes | Pembrolizumab 2 mg/kg Q3W | Chemotherapy | 62/63 | 58/64 | 349 (178/171) |
| Hamid 2 2017 | NCT01704287 | Melanoma | Yes | Pembrolizumab 10 mg/kg Q3W | Chemotherapy | 60/63 | 60/64 | 350 (179/171) |
| Pelosof 2021 | NCT02569242 | Esophageal squamous cell carcinoma | Yes | Nivolumab 240mg Q2W | Chemotherapy | 62.8/64.9 | 85/89 | 417 (209/208) |
| Kelly 2021 | NCT02743494 | Esophageal or Gastroesophageal Junction Cancer | Yes | Nivolumab 240mg Q2W | Placebo | 62/61 | 84/85 | 792 (532/260) |
| Wu 2019 | NCT02613507 | NSCLC | Yes | Nivolumab 3mg/kg Q2W | Chemotherapy | 60/60 | 78/81 | 493 (337/156) |
| Hellmann 2018 | NCT02477826 | NSCLC | No | Nivolumab 240mg Q2W | Chemotherapy | 64/64 | 69/66 | 961 (391/570) |
| Ferris 2016 | NCT02105636 | Head-and-neck squamous cell carcinoma | Yes | Nivolumab 3mg/kg Q2W | Chemotherapy | 59/61 | 82.1/85.1 | 347 (236/111) |
| Robert 2015 | NCT01721772 | Melanoma | No | Nivolumab 3mg/kg Q2W | Chemotherapy | 64/66 | 57.6/60.1 | 411 (206/205) |
| Brahmer 2015 | NCT01642004 | NSCLC | Yes | Nivolumab 3mg/kg Q2W | Chemotherapy | 62/64 | 82/71 | 260 (131/129) |
| Borghaei 2015 | NCT01673867 | NSCLC | Yes | Nivolumab 3mg/kg Q2W | Chemotherapy | 61/64 | 52/58 | 555 (287/268) |
| Kang 2017 | NCT02267343 | Gastric or  gastro-oesophageal junction cancer | Yes | Nivolumab 3mg/kg Q2W | Placebo | 62/61 | 69/73 | 491 (330/161) |
| Carbone 2017 | NCT02041533 | NSCLC | No | Nivolumab 3mg/kg Q2W | Chemotherapy | 63/65 | 68/45 | 530 (267/263) |
| Zimmer 2020 | NCT02523313 | Melanoma | Yes | Nivolumab 3mg/kg Q2W | Placebo | 57/58.5 | 53/63 | 107 (56/51) |
| Larkin 2018 | NCT01721746 | Melanoma | Yes | Nivolumab 3mg/kg Q2W | Chemotherapy | 59/62 | 65/64 | 370 (268/102) |
| Fennell 2021 | NCT03063450 | Malignant mesothelioma | Yes | Nivolumab 240mg Q2W | Placebo | 70/71 | 76/78 | 332 (221/111) |
| Hamanishi 2021 | JapicCTI-153004 | Ovarian cancer | Yes | Nivolumab 240mg Q2W | Chemotherapy | 58/60 | 0/0 | 311 (156/155) |
| Reardon 2020 | NCT02017717 | Glioblastoma | Yes | Nivolumab 3mg/kg Q2W | Targeted therapy | 55.5/55 | 63/64.3 | 347 (182/165) |
| Bajorin 2021 | NCT02632409 | Urothelial carcinoma | Yes | Nivolumab 240mg Q2W | Placebo | 65.3/65.9 | 75.1/7.2 | 699 (351/348) |
| Shen 2022 | NCT03430843 | Esophageal squamous cell carcinoma | Yes | Tislelizumab 200mg Q3W | Chemotherapy | 62/63 | 84.8/84 | 495 (255/240) |
| Zhou 2023 | NCT03358875 | NSCLC | Yes | Tislelizumab 200mg Q3W | Chemotherapy | 61/61 | 77.8/76.3 | 792 (534/258) |
| Xu 2022 | NCT03116152 | Esophageal squamous cell carcinoma | Yes | Sintilimab 200 mg Q3W | Chemotherapy | 60/60 | 92.6/88.4 | 181 (94/87) |
| Shi 2022 | NCT03150875 | NSCLC | Yes | Sintilimab 200 mg Q3W | Chemotherapy | 61/60 | 93.8/90.4 | 274 (144/130) |
| Huang 2020 | NCT03099382 | Esophageal squamous cell carcinoma | Yes | Camrelizumab 200 mg Q2W | Chemotherapy | 60/60 | 91/87 | 448 (228/220) |
| Lian 2022 | NCT03178123 | Melanoma | Yes | Toripalimab 3 mg/kg Q2W | Interferon | 57.4/57.7 | 30.1/41.7 | 145 (73/72) |

I/C: intervention/control.

**Table S3 Characteristics of the included studies for hyperthyroidism.**

| **Author/year** | **RCT number** | **Tumor types** | **Previous treatment** | **Intervention** | **Control** | **Age**  **(year, I/C)** | **Gender**  **(%male, I/C)** | **Sample size (I/C)** |
| --- | --- | --- | --- | --- | --- | --- | --- | --- |
| Herbst 1 2016 | NCT01905657 | NSCLC | Yes | Pembrolizumab 2 mg/kg Q3W | Chemotherapy | 63/62 | 62/61 | 648 (339/309) |
| Herbst 2 2016 | NCT01905657 | NSCLC | Yes | Pembrolizumab 10 mg/kg Q3W | Chemotherapy | 63/62 | 62/61 | 652 (343/309) |
| Bellmunt 2017 | NCT02256436 | Urothelial carcinoma | Yes | Pembrolizumab 200 mg Q3W | Chemotherapy | 67/65 | 74.1/74.3 | 521 (266/255) |
| Cohen 2019 | NCT02252042 | Head-and-neck squamous cell carcinoma | Yes | Pembrolizumab 200 mg Q3W | Chemotherapy | 60/60 | 84/83 | 480 (246/234) |
| Eggermont 2018 | NCT02362594 | Melanoma | Yes | Pembrolizumab 200 mg Q3W | Placebo | 54/54 | 63/60.2 | 1011 (509/502) |
| Finn 2020 | NCT02702401 | Hepatocellular carcinoma | Yes | Pembrolizumab 200 mg Q3W | Placebo | 67/65 | 81.3/83 | 413 (279/134) |
| Shitara 2018 | NCT02370498 | Gastric or gastrooesophageal junction cancer | Yes | Pembrolizumab 200 mg Q3W | Chemotherapy | 62.5/60 | 68/70 | 570 (294/276) |
| Kuruvilla 2021 | NCT02684292 | Hodgkin lymphoma | Yes | Pembrolizumab 200 mg Q3W | Targeted therapy | 36/35 | 56/59 | 300 (148/152) |
| Shitara 2020 | NCT02494583 | Gastric Cancer | No | Pembrolizumab 200 mg Q3W | Chemotherapy | 61/62.5 | 70.3/71.6 | 498 (254/244) |
| Winer 2021 | NCT02555657 | Metastatic triple-negative breast cancer | Yes | Pembrolizumab 200 mg Q3W | Chemotherapy | 50/53 | 0/1 | 601 (309/292) |
| André 2020 | NCT02563002 | Colorectal cancer | Yes | Pembrolizumab 200 mg Q3W | Chemotherapy | 63/62.5 | 46/53 | 296 (153/143) |
| Kojima 2020 | NCT02564263 | Esophageal cancer | Yes | Pembrolizumab 200 mg Q3W | Chemotherapy | 63/62 | 86.9/86.3 | 610 (314/296) |
| Burtness 2019 | NCT02358031 | Head-and-neck squamous cell carcinoma | No | Pembrolizumab 200 mg Q3W | Chemotherapy | 62/61 | 83/87 | 587 (300/287) |
| Qin 2023 | NCT03062358 | Hepatocellular carcinoma | Yes | Pembrolizumab 200 mg Q3W | Placebo | 54/54 | 85.7/82.4 | 452 (299/153) |
| Powles 2022 | NCT03142334 | Clear cell renal cell carcinoma | No | Pembrolizumab 200 mg Q3W | Placebo | 60/60 | 70/72 | 984 (488/496) |
| Long 2022 | NCT03553836 | Melanoma | No | Pembrolizumab 200 mg Q3W | Placebo | 60/61 | 62/59 | 969 (483/486) |
| Chung 2022 | NCT03019588 | Gastric or gastroesophageal junction cancer | Yes | Pembrolizumab 200 mg Q3W | Chemotherapy | 61/61 | 68/79 | 91 (47/44) |
| Chan 2023 | NCT02611960 | Nasopharyngeal cancer | Yes | Pembrolizumab 200 mg Q3W | Chemotherapy | 51/53 | 83.8/81.9 | 228 (116/112) |
| Popat 2020 | NCT02991482 | Malignant pleural mesothelioma | Yes | Pembrolizumab 200 mg Q3W | Chemotherapy | 70/69 | 81.9/79.4 | 142 (72/70) |
| Ren 2023 | NCT02864394 | NSCLC | Yes | Pembrolizumab 2 mg/kg Q3W | Chemotherapy | 61/61 | 73.7/77.4 | 411 (213/198) |
| Reck 2021 | NCT02142738 | NSCLC | No | Pembrolizumab 200 mg Q3W | Chemotherapy | 64.5/66 | 59.7/62.9 | 304 (154/150) |
| Castro 2023 | NCT02220894 | NSCLC | No | Pembrolizumab 200 mg Q3W | Chemotherapy | 63/63 | 71/71 | 1251 (636/615) |
| Hamid 1 2017 | NCT01704287 | Melanoma | Yes | Pembrolizumab 2 mg/kg Q3W | Chemotherapy | 62/63 | 58/64 | 349 (178/171) |
| Hamid 2 2017 | NCT01704287 | Melanoma | Yes | Pembrolizumab 10 mg/kg Q3W | Chemotherapy | 60/63 | 60/64 | 350 (179/171) |
| Kelly 2021 | NCT02743494 | Esophageal or gastroesophageal junction Cancer | Yes | Nivolumab 240mg Q2W | Placebo | 62/61 | 84/85 | 792 (532/260) |
| Wu 2019 | NCT02613507 | NSCLC | Yes | Nivolumab 3mg/kg Q2W | Chemotherapy | 60/60 | 78/81 | 493 (337/156) |
| Ferris 2016 | NCT02105636 | Head-and-neck squamous cell carcinoma | Yes | Nivolumab 3mg/kg Q2W | Chemotherapy | 59/61 | 82.1/85.1 | 347 (236/111) |
| Robert 2015 | NCT01721772 | Melanoma | No | Nivolumab 3mg/kg Q2W | Chemotherapy | 64/66 | 57.6/60.1 | 411 (206/205) |
| Borghaei 2015 | NCT01673867 | NSCLC | Yes | Nivolumab 3mg/kg Q2W | Chemotherapy | 61/64 | 52/58 | 555 (287/268) |
| Kang 2017 | NCT02267343 | Gastric or gastro-oesophageal junction cancer | Yes | Nivolumab 3mg/kg Q2W | Placebo | 62/61 | 69/73 | 491 (330/161) |
| Zimmer 2020 | NCT02523313 | Melanoma | Yes | Nivolumab 3mg/kg Q2W | Placebo | 57/58.5 | 53/63 | 107 (56/51) |
| Larkin 2018 | NCT01721746 | Melanoma | Yes | Nivolumab 3mg/kg Q2W | Chemotherapy | 59/62 | 65/64 | 370 (268/102) |
| Hamanishi 2021 | JapicCTI-153004 | Ovarian cancer | Yes | Nivolumab 240mg Q2W | Chemotherapy | 58/60 | 0/0 | 311 (156/155) |
| Reardon 2020 | NCT02017717 | Glioblastoma | Yes | Nivolumab 3mg/kg Q2W | Targeted therapy | 55.5/55 | 63/64.3 | 347 (182/165) |
| Bajorin 2021 | NCT02632409 | Urothelial carcinoma | Yes | Nivolumab 240mg Q2W | Placebo | 65.3/65.9 | 75.1/77.2 | 699 (351/348) |
| Shi 2022 | NCT03150875 | NSCLC | Yes | Sintilimab 200 mg Q3W | Chemotherapy | 61/60 | 93.8/90.4 | 274 (144/130) |
| Lian 2022 | NCT03178123 | Melanoma | Yes | Toripalimab 3 mg/kg Q2W | Interferon | 57.4/57.7 | 30.1/41.7 | 145 (73/72) |

I/C: intervention/control.

**Table S4 Characteristics of the included studies for thyroiditis.**

| **Author / year** | **RCT number** | **Tumor types** | **Previous treatment** | **Intervention** | **Control** | **Age**  **(year, I/C)** | **Gender**  **(%male, I/C)** | **Sample size**  **(I/C)** |
| --- | --- | --- | --- | --- | --- | --- | --- | --- |
| Herbst 1 2016 | NCT01905657 | NSCLC | Yes | Pembrolizumab 2 mg/kg Q3W | Chemotherapy | 63/62 | 62/61 | 648 (339/309) |
| Bellmunt 2017 | NCT02256436 | Urothelial carcinoma | Yes | Pembrolizumab 200 mg Q3W | Chemotherapy | 67/65 | 74.1/74.3 | 521 (266/255) |
| Eggermont 2018 | NCT02362594 | Melanoma | Yes | Pembrolizumab 200 mg Q3W | Placebo | 54/54 | 63/60.2 | 1011 (509/502) |
| Finn 2020 | NCT02702401 | Hepatocellular carcinoma | Yes | Pembrolizumab 200 mg Q3W | Placebo | 67/65 | 81.3/83 | 413 (279/134) |
| Shitara 2020 | NCT02494583 | Gastric cancer | No | Pembrolizumab 200 mg Q3W | Chemotherapy | 61/62.5 | 70.3/71.6 | 498 (254/244) |
| Winer 2021 | NCT02555657 | Metastatic triple-negative breast cancer | Yes | Pembrolizumab 200 mg Q3W | Chemotherapy | 50/53 | 0/1 | 601 (309/292) |
| André 2020 | NCT02563002 | Colorectal cancer | Yes | Pembrolizumab 200 mg Q3W | Chemotherapy | 63/62.5 | 46/53 | 296 (153/143) |
| Kojima 2020 | NCT02564263 | Esophageal cancer | Yes | Pembrolizumab 200 mg Q3W | Chemotherapy | 63/62 | 86.9/86.3 | 610 (314/296) |
| Powles 2022 | NCT03142334 | Clear cell renal cell carcinoma | No | Pembrolizumab 200 mg Q3W | Placebo | 60/60 | 70/72 | 984 (488/496) |
| Long 2022 | NCT03553836 | Melanoma | No | Pembrolizumab 200 mg Q3W | Placebo | 60/61 | 62/59 | 969 (483/486) |
| Chung 2022 | NCT03019588 | Gastric or gastroesophageal junction cancer | Yes | Pembrolizumab 200 mg Q3W | Chemotherapy | 61/61 | 68/79 | 91 (47/44) |
| Reck 2021 | NCT02142738 | NSCLC | No | Pembrolizumab 200 mg Q3W | Chemotherapy | 64.5/66 | 59.7/62.9 | 304 (154/150) |
| Castro 2023 | NCT02220894 | NSCLC | No | Pembrolizumab 200 mg Q3W | Chemotherapy | 63/63 | 71/71 | 1251 (636/615) |
| Wu 2019 | NCT02613507 | NSCLC | Yes | Nivolumab 3mg/kg Q2W | Chemotherapy | 60/60 | 78/81 | 493 (337/156) |
| Ferris 2016 | NCT02105636 | Head-and-neck squamous cell carcinoma | Yes | Nivolumab 3mg/kg Q2W | Chemotherapy | 59/61 | 82.1/85.1 | 347 (236/111) |
| Borghaei 2015 | NCT01673867 | NSCLC | Yes | Nivolumab 3mg/kg Q2W | Chemotherapy | 61/64 | 52/58 | 555 (287/268) |
| Kang 2017 | NCT02267343 | Gastric or gastro-oesophageal junction cancer | Yes | Nivolumab 3mg/kg Q2W | Placebo | 62/61 | 69/73 | 491 (330/161) |
| Zimmer 2020 | NCT02523313 | melanoma | Yes | Nivolumab 3mg/kg Q2W | Placebo | 57/58.5 | 53/63 | 107 (56/51) |
| Bajorin 2021 | NCT02632409 | Urothelial carcinoma | Yes | Nivolumab 240mg Q2W | Placebo | 65.3/65.9 | 75.1/77.2 | 699 (351/348) |

I/C: intervention/control.

**Table S5 Characteristics of the included studies for hypophysitis.**

| **Author / year** | **RCT number** | **Tumor types** | **Previous treatment** | **Intervention** | **Control** | **Age**  **(year, I/C)** | **Gender (%male, I/C)** | **Sample size**  **(I/C)** |
| --- | --- | --- | --- | --- | --- | --- | --- | --- |
| Herbst 1 2016 | NCT01905657 | NSCLC | Yes | Pembrolizumab 2 mg/kg Q3W | Chemotherapy | 63/62 | 62/61 | 648 (339/309) |
| Herbst 2 2016 | NCT01905657 | NSCLC | Yes | Pembrolizumab 10 mg/kg Q3W | Chemotherapy | 63/62 | 62/61 | 652 (343/309) |
| Eggermont 2018 | NCT02362594 | Melanoma | Yes | Pembrolizumab 200 mg Q3W | Placebo | 54/54 | 63/60.2 | 1011 (509/502) |
| Finn 2020 | NCT02702401 | Hepatocellular carcinoma | Yes | Pembrolizumab 200 mg Q3W | Placebo | 67/65 | 81.3/83 | 413 (279/134) |
| Shitara 2018 | NCT02370498 | Gastric or gastrooesophageal junction cancer | Yes | Pembrolizumab 200 mg Q3W | Chemotherapy | 62.5/60 | 68/70 | 570 (294/276) |
| Shitara 2020 | NCT02494583 | Gastric cancer | No | Pembrolizumab 200 mg Q3W | Chemotherapy | 61/62.5 | 70.3/71.6 | 498 (254/244) |
| André 2020 | NCT02563002 | Colorectal cancer | Yes | Pembrolizumab 200 mg Q3W | Chemotherapy | 63/62.5 | 46/53 | 296 (153/143) |
| Kojima 2020 | NCT02564263 | Esophageal cancer | Yes | Pembrolizumab 200 mg Q3W | Chemotherapy | 63/62 | 86.9/86.3 | 610 (314/296) |
| Burtness 2019 | NCT02358031 | Head-and-neck squamous cell carcinoma | No | Pembrolizumab 200 mg Q3W | Chemotherapy | 62/61 | 83/87 | 587 (300/287) |
| Qin 2023 | NCT03062358 | Hepatocellular carcinoma | Yes | Pembrolizumab 200 mg Q3W | Placebo | 54/54 | 85.7/82.4 | 452 (299/153) |
| Powles 2022 | NCT03142334 | Clear cell renal cell carcinoma | No | Pembrolizumab 200 mg Q3W | Placebo | 60/60 | 70/72 | 984 (488/496) |
| Long 2022 | NCT03553836 | Melanoma | No | Pembrolizumab 200 mg Q3W | Placebo | 60/61 | 62/59 | 969 (483/486) |
| Popat 2020 | NCT02991482 | Malignant pleural mesothelioma | Yes | Pembrolizumab 200 mg Q3W | Chemotherapy | 70/69 | 81.9/79.4 | 142 (72/70) |
| Reck 2021 | NCT02142738 | NSCLC | No | Pembrolizumab 200 mg Q3W | Chemotherapy | 64.5/66 | 59.7/62.9 | 304 (154/150) |
| Castro 2023 | NCT02220894 | NSCLC | No | Pembrolizumab 200 mg Q3W | Chemotherapy | 63/63 | 71/71 | 1251 (636/615) |
| Hamid 1 2017 | NCT01704287 | Melanoma | Yes | Pembrolizumab 2 mg/kg Q3W | Chemotherapy | 62/63 | 58/64 | 349 (178/171) |
| Hamid 2 2017 | NCT01704287 | Melanoma | Yes | Pembrolizumab 10 mg/kg Q3W | Chemotherapy | 60/63 | 60/64 | 350 (179/171) |
| Ferris 2016 | NCT02105636 | Head-and-neck squamous cell carcinoma | Yes | Nivolumab 3mg/kg Q2W | Chemotherapy | 59/61 | 82.1/85.1 | 347 (236/111) |
| Robert 2015 | NCT01721772 | Melanoma | No | Nivolumab 3mg/kg Q2W | Chemotherapy | 64/66 | 57.6/60.1 | 411 (206/205) |
| Kang 2017 | NCT02267343 | Gastric or gastro-oesophageal junction cancer | Yes | Nivolumab 3mg/kg Q2W | Placebo | 62/61 | 69/73 | 491 (330/161) |
| Zimmer 2020 | NCT02523313 | Melanoma | Yes | Nivolumab 3mg/kg Q2W | Placebo | 57/58.5 | 53/63 | 107 (56/51) |
| Yau 2022 | NCT02576509 | Hepatocellular carcinoma | No | Nivolumab 240mg Q2W | Targeted therapy | 65/65 | 85/85 | 730 (367/363) |

I/C: intervention/control.

**Table S6 Characteristics of the included studies for adrenal insufficiency.**

| **Author / year** | **RCT number** | **Tumor types** | **Previous treatment** | **Intervention** | **Control** | **Age**  **(year, I/C)** | **Gender (%male, I/C)** | **Sample size (I/C)** |
| --- | --- | --- | --- | --- | --- | --- | --- | --- |
| Herbst 1 2016 | NCT01905657 | NSCLC | Yes | Pembrolizumab 2 mg/kg Q3W | Chemotherapy | 63/62 | 62/61 | 648 (339/309) |
| Herbst 2 2016 | NCT01905657 | NSCLC | Yes | Pembrolizumab 10 mg/kg Q3W | Chemotherapy | 63/62 | 62/61 | 652 (343/309) |
| Bellmunt 2017 | NCT02256436 | Urothelial carcinoma | Yes | Pembrolizumab 200 mg Q3W | Chemotherapy | 67/65 | 74.1/74.3 | 521 (266/255) |
| Eggermont 2018 | NCT02362594 | Melanoma | Yes | Pembrolizumab 200 mg Q3W | Placebo | 54/54 | 63/60.2 | 1011 (509/502) |
| Finn 2020 | NCT02702401 | Hepatocellular carcinoma | Yes | Pembrolizumab 200 mg Q3W | Placebo | 67/65 | 81.3/83 | 413 (279/134) |
| Shitara 2020 | NCT02494583 | Gastric cancer | No | Pembrolizumab 200 mg Q3W | Chemotherapy | 61/62.5 | 70.3/71.6 | 498 (254/244) |
| Winer 2021 | NCT02555657 | Metastatic triple-negative breast cancer | Yes | Pembrolizumab 200 mg Q3W | Chemotherapy | 50/53 | 0/1 | 601 (309/292) |
| André 2020 | NCT02563002 | Colorectal cancer | Yes | Pembrolizumab 200 mg Q3W | Chemotherapy | 63/62.5 | 46/53 | 296 (153/143) |
| Burtness 2019 | NCT02358031 | Head-and-neck squamous cell carcinoma | No | Pembrolizumab 200 mg Q3W | Chemotherapy | 62/61 | 83/87 | 587 (300/287) |
| Powles 2022 | NCT03142334 | Clear cell renal cell carcinoma | No | Pembrolizumab 200 mg Q3W | Placebo | 60/60 | 70/72 | 984 (488/496) |
| Long 2022 | NCT03553836 | Melanoma | No | Pembrolizumab 200 mg Q3W | Placebo | 60/61 | 62/59 | 969 (483/486) |
| Chung 2022 | NCT03019588 | Gastric or gastroesophageal junction cancer | Yes | Pembrolizumab 200 mg Q3W | Chemotherapy | 61/61 | 68/79 | 91 (47/44) |
| Chan 2023 | NCT02611960 | Nasopharyngeal cancer | Yes | Pembrolizumab 200 mg Q3W | Chemotherapy | 51/53 | 83.8/81.9 | 228 (116/112) |
| Popat 2020 | NCT02991482 | Malignant pleural mesothelioma | Yes | Pembrolizumab 200 mg Q3W | Chemotherapy | 70/69 | 81.9/79.4 | 142 (72/70) |
| Castro 2023 | NCT02220894 | NSCLC | No | Pembrolizumab 200 mg Q3W | Chemotherapy | 63/63 | 71/71 | 1251 (636/615) |
| Hellmann 2018 | NCT02477826 | NSCLC | No | Nivolumab 240mg Q2W | Chemotherapy | 64/64 | 69/66 | 961 (391/570) |
| Ferris 2016 | NCT02105636 | Head-and-neck squamous cell carcinoma | Yes | Nivolumab 3mg/kg Q2W | Chemotherapy | 59/61 | 82.1/85.1 | 347 (236/111) |
| Yau 2022 | NCT02576509 | Hepatocellular Carcinoma | No | Nivolumab 240mg Q2W | Targeted therapy | 65/65 | 85/85 | 730 (367/363) |
| Hamanishi 2021 | JapicCTI-153004 | Ovarian cancer | Yes | Nivolumab 240mg Q2W | Chemotherapy | 58/60 | 0/0 | 311 (156/155) |
| Bajorin 2021 | NCT02632409 | Urothelial carcinoma | Yes | Nivolumab 240mg Q2W | Placebo | 65.3/65.9 | 75.1/77.2 | 699 (351/348) |
| Huang 2020 | NCT03099382 | Esophageal squamous cell carcinoma | Yes | Camrelizumab 200 mg Q2W | Chemotherapy | 60/60 | 91/87 | 448 (228/220) |

I/C: intervention/control.

**Table S7 Characteristics of the included studies for diabetes mellitus.**

| **Author / year** | **RCT number** | **Tumor types** | **Previous treatment** | **Intervention** | **Control** | **Age (year, I/C)** | **Gender**  **(%male, I/C)** | **Sample size**  **(I/C)** |
| --- | --- | --- | --- | --- | --- | --- | --- | --- |
| Herbst 1 2016 | NCT01905657 | NSCLC | Yes | Pembrolizumab 2 mg/kg Q3W | Chemotherapy | 63/62 | 62/61 | 648 (339/309) |
| Herbst 2 2016 | NCT01905657 | NSCLC | Yes | Pembrolizumab 10 mg/kg Q3W | Chemotherapy | 63/62 | 62/61 | 652 (343/309) |
| Eggermont 2018 | NCT02362594 | Melanoma | Yes | Pembrolizumab 200 mg Q3W | Placebo | 54/54 | 63/60.2 | 1011 (509/502) |
| Finn 2020 | NCT02702401 | Hepatocellular carcinoma | Yes | Pembrolizumab 200 mg Q3W | Placebo | 67/65 | 81.3/83 | 413 (279/134) |
| Shitara 2018 | NCT02370498 | Gastric or gastrooesophageal junction cancer | Yes | Pembrolizumab 200 mg Q3W | Chemotherapy | 62.5/60 | 68/70 | 570 (294/276) |
| Shitara 2020 | NCT02494583 | Gastric cancer | No | Pembrolizumab 200 mg Q3W | Chemotherapy | 61/62.5 | 70.3/71.6 | 498 (254/244) |
| Winer 2021 | NCT02555657 | Metastatic triple-negative breast cancer | Yes | Pembrolizumab 200 mg Q3W | Chemotherapy | 50/53 | 0/1 | 601 (309/292) |
| André 2020 | NCT02563002 | Colorectal cancer | Yes | Pembrolizumab 200 mg Q3W | Chemotherapy | 63/62.5 | 46/53 | 296 (153/143) |
| Kojima 2020 | NCT02564263 | Esophageal cancer | Yes | Pembrolizumab 200 mg Q3W | Chemotherapy | 63/62 | 86.9/86.3 | 610 (314/296) |
| Powles 2022 | NCT03142334 | Clear cell renal cell carcinoma | No | Pembrolizumab 200 mg Q3W | Placebo | 60/60 | 70/72 | 984 (488/496) |
| Long 2022 | NCT03553836 | Melanoma | No | Pembrolizumab 200 mg Q3W | Placebo | 60/61 | 62/59 | 969 (483/486) |
| Reck 2021 | NCT02142738 | NSCLC | No | Pembrolizumab 200 mg Q3W | Chemotherapy | 64.5/66 | 59.7/62.9 | 304 (154/150) |
| Wu 2019 | NCT02613507 | NSCLC | Yes | Nivolumab 3mg/kg Q2W | Chemotherapy | 60/60 | 78/81 | 493 (337/156) |
| Robert 2015 | NCT01721772 | Melanoma | No | Nivolumab 3mg/kg Q2W | Chemotherapy | 64/66 | 57.6/60.1 | 411 (206/205) |
| Borghaei 2015 | NCT01673867 | NSCLC | Yes | Nivolumab 3mg/kg Q2W | Chemotherapy | 61/64 | 52/58 | 555 (287/268) |
| Kang 2017 | NCT02267343 | Gastric or gastro-oesophageal junction cancer | Yes | Nivolumab 3mg/kg Q2W | Placebo | 62/61 | 69/73 | 491 (330/161) |
| Zimmer 2020 | NCT02523313 | Melanoma | Yes | Nivolumab 3mg/kg Q2W | Placebo | 57/58.5 | 53/63 | 107 (56/51) |
| Bajorin 2021 | NCT02632409 | Urothelial carcinoma | Yes | Nivolumab 240mg Q2W | Placebo | 65.3/65.9 | 75.1/77.2 | 699 (351/348) |
| Huang 2020 | NCT03099382 | Esophageal squamous cell carcinoma | Yes | Camrelizumab 200 mg Q2W | Chemotherapy | 60/60 | 91/87 | 448 (228/220) |

I/C: intervention/control.

**^
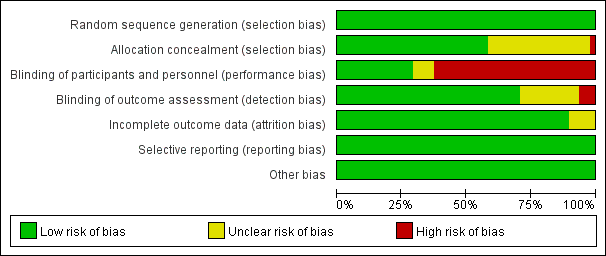
^**

**Figure S1.** Risk of bias item presented as percentages across all included studies.

**
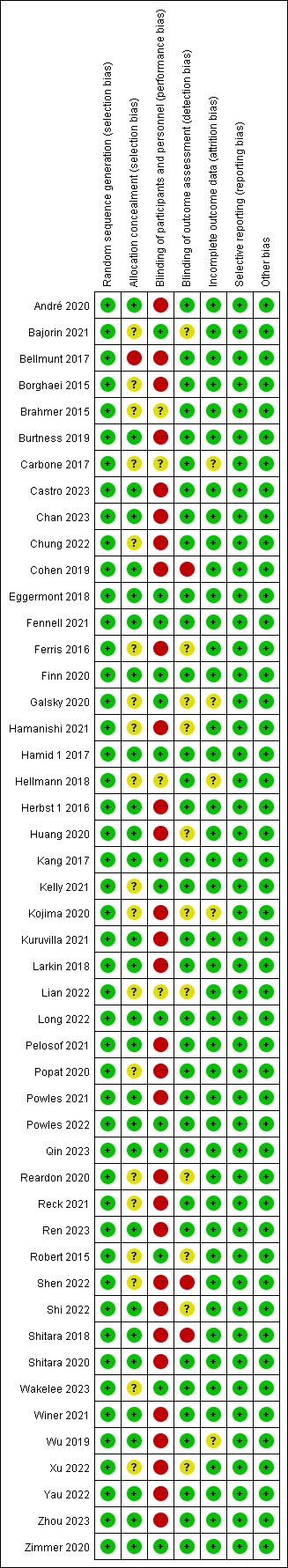
**

**Figure S2.** Risk of bias summary and graph.

**
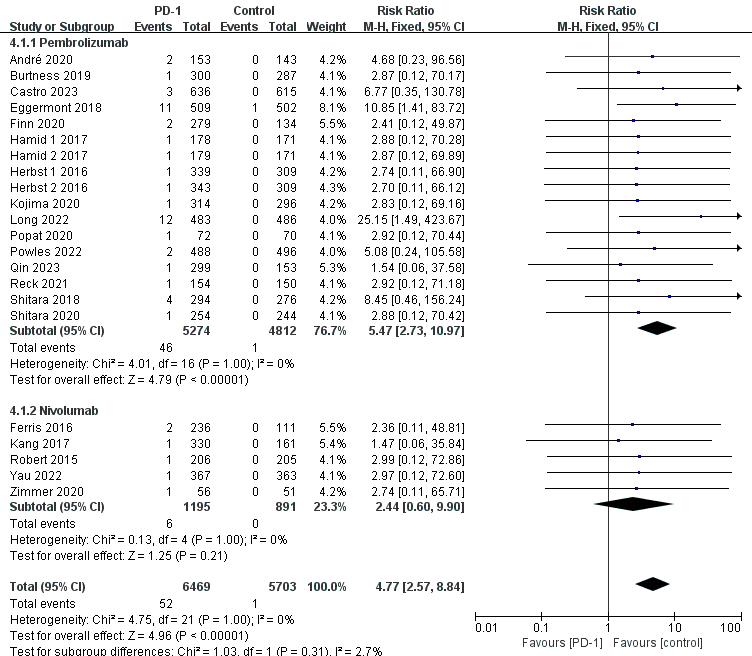
**

**Figure S3.** Forest plots of the relative risks of hypophysitis related to different PD-1 inhibitors.

**
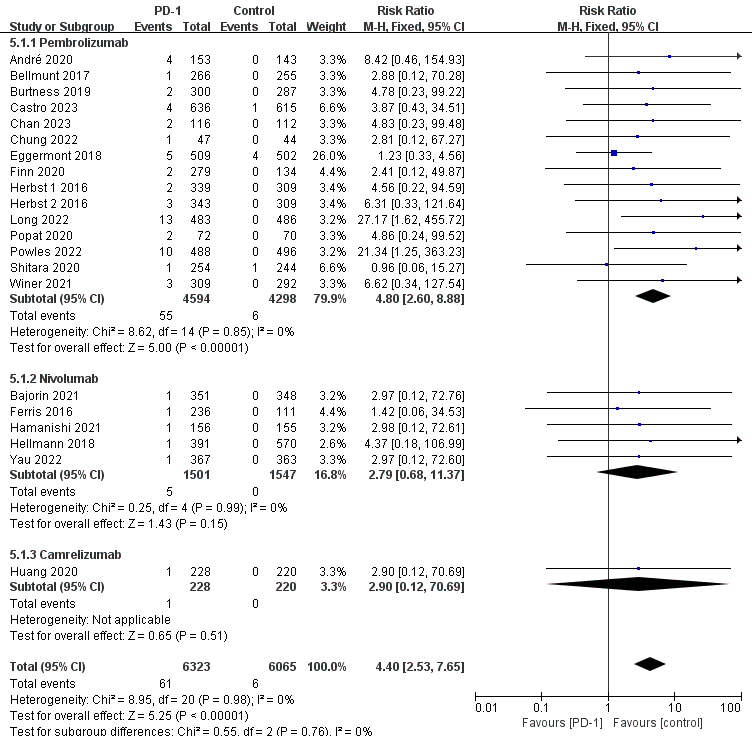
**

**Figure S4.** Forest plots of the relative risks of adrenal insufficiency related to different PD-1 inhibitors.

**
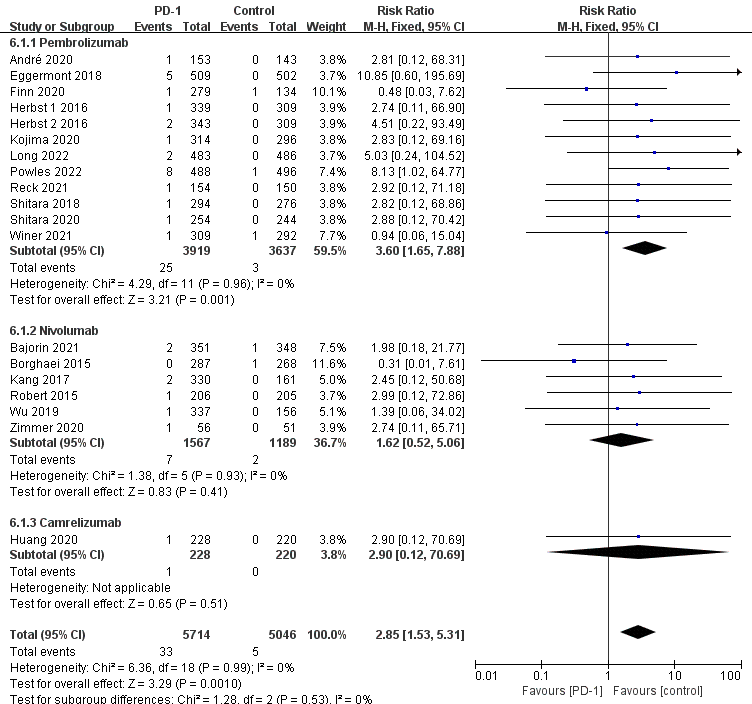
**

**Figure S5.** Forest plots of the relative risks of diabetes mellitus related to different PD-1 inhibitors.

**
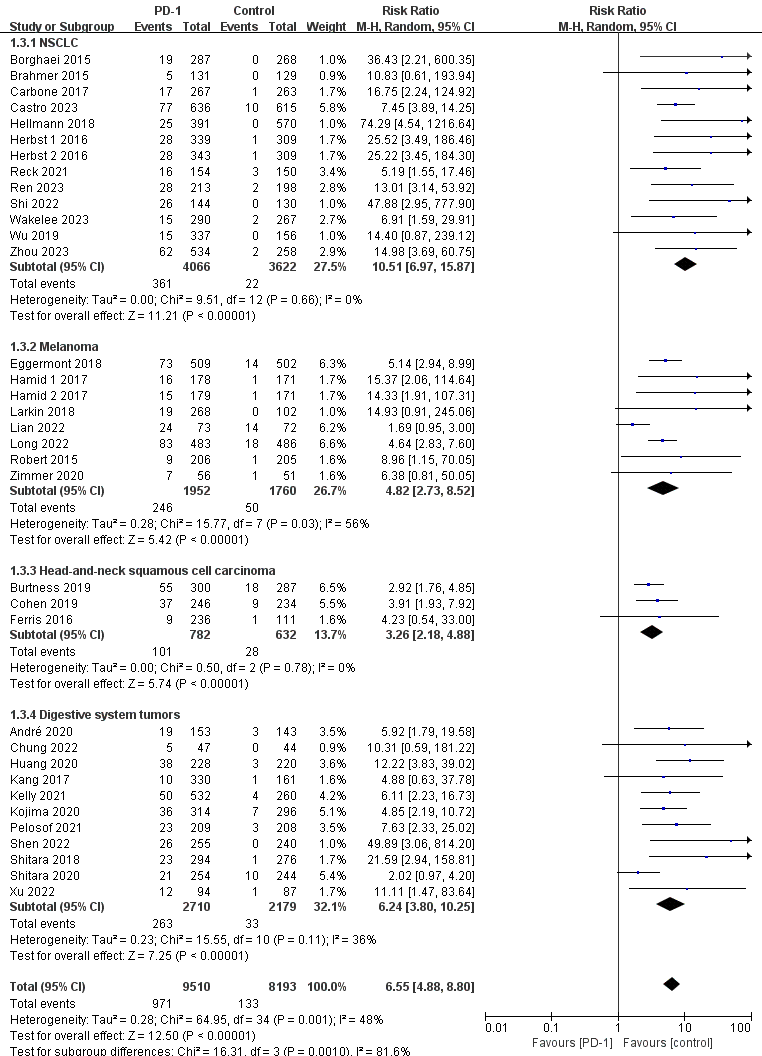
**

**Figure S6.** Forest plots of the relative risks of hypothyroidism related to different tumor types.

**
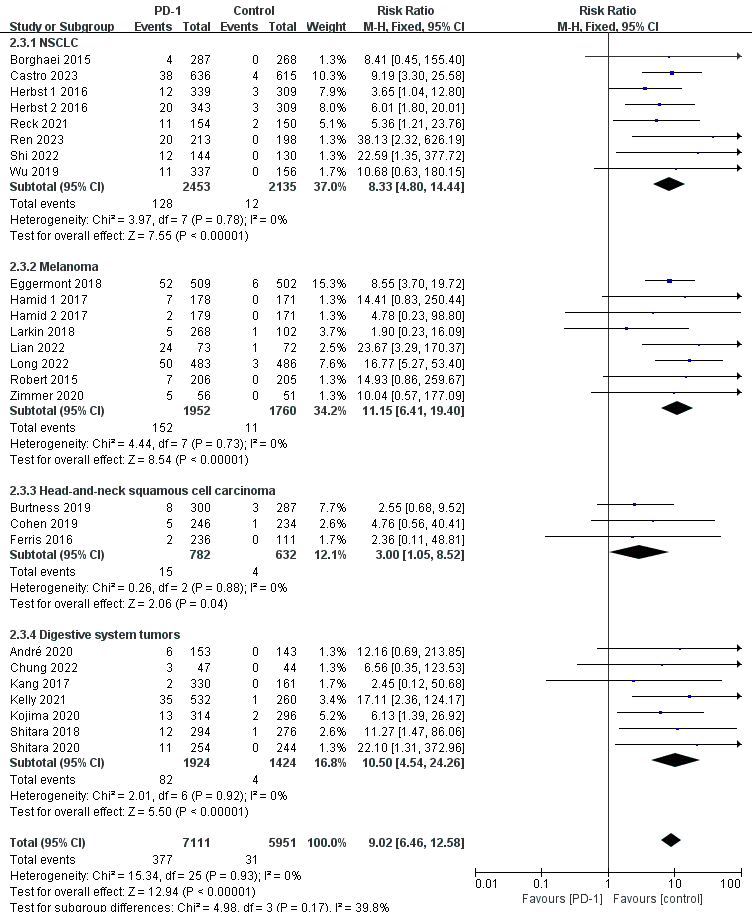
**

**Figure S7.** Forest plots of the relative risks of hyperthyroidism related to different tumor types.

**
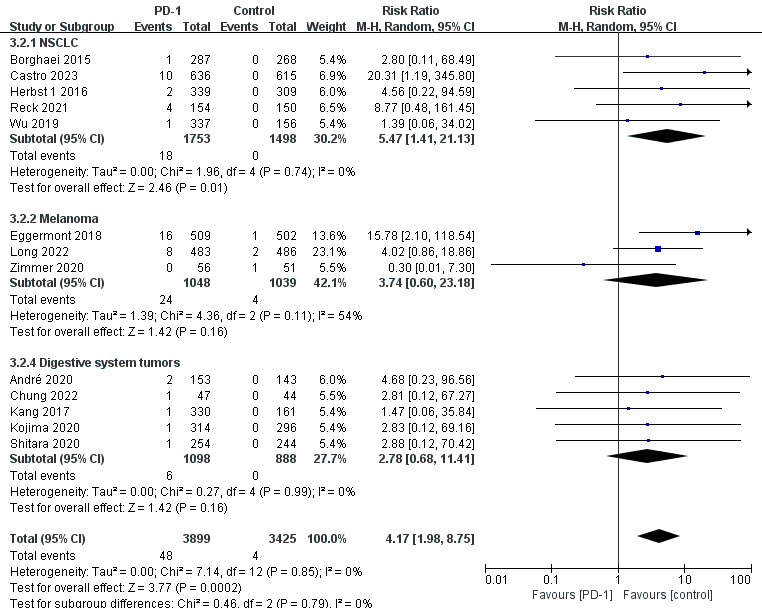
**

**Figure S8.** Forest plots of the relative risks of thyroiditis related to different tumor types.

**
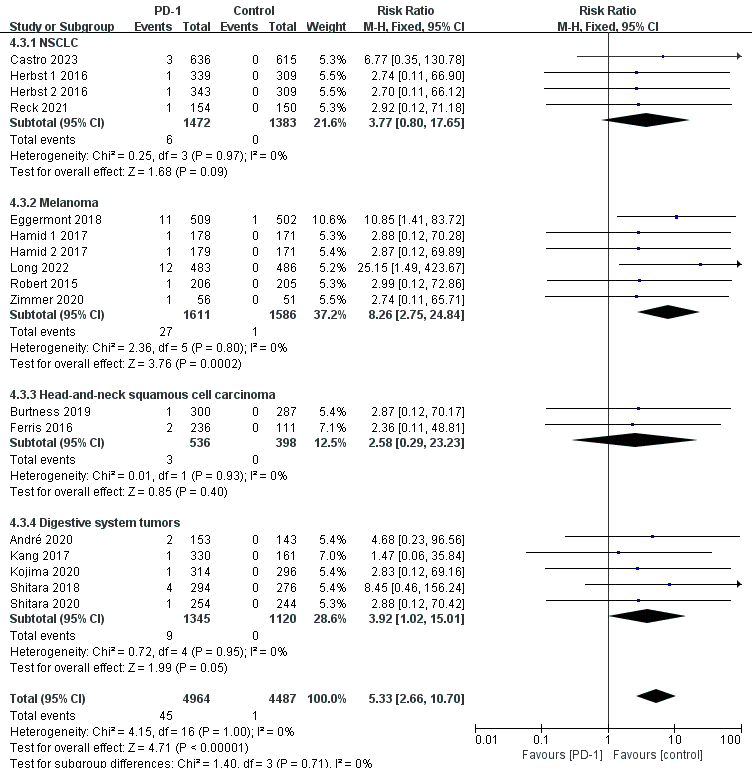
**

**Figure S9.** Forest plots of the relative risks of hypophysitis related to different tumor types.

**
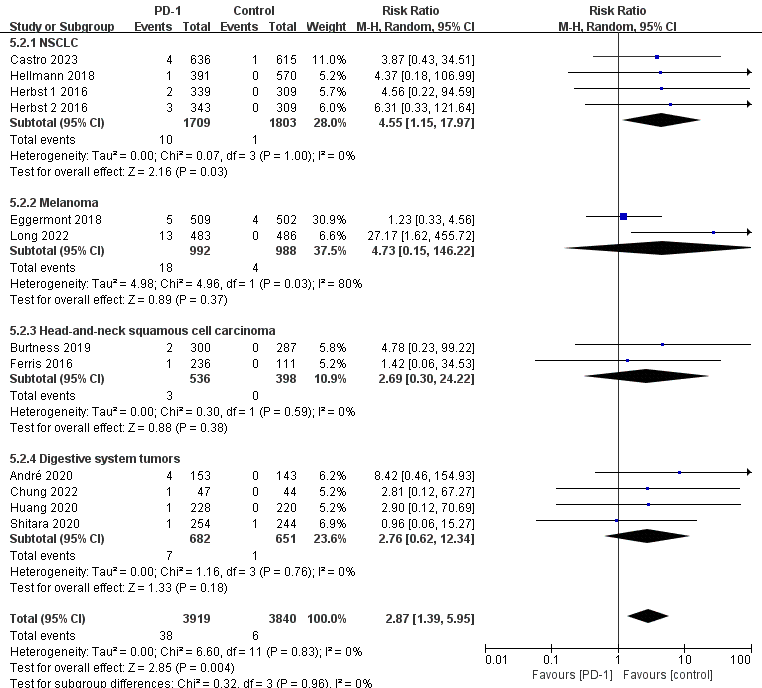
**

**Figure S10.** Forest plots of the relative risks of adrenal insufficiency related to different tumor types.

**
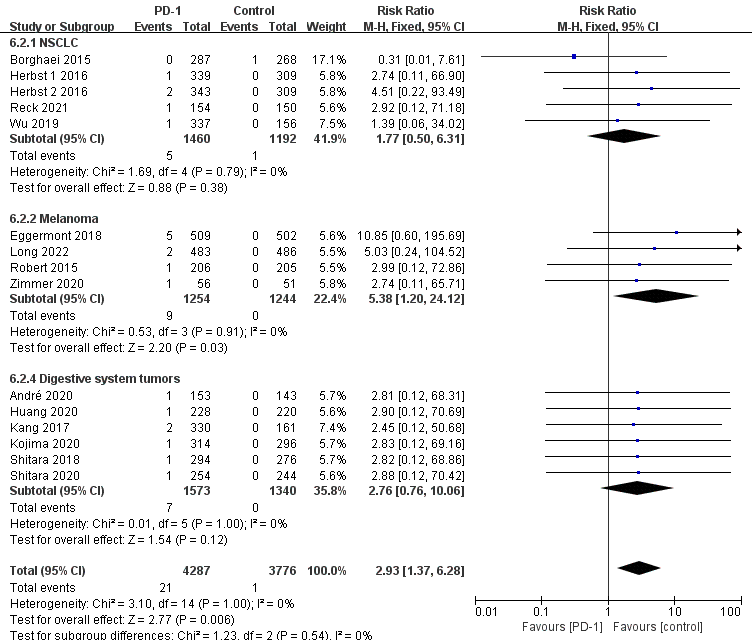
**

**Figure S11.** Forest plots of the relative risks of diabetes mellitus related to different tumor types.

**
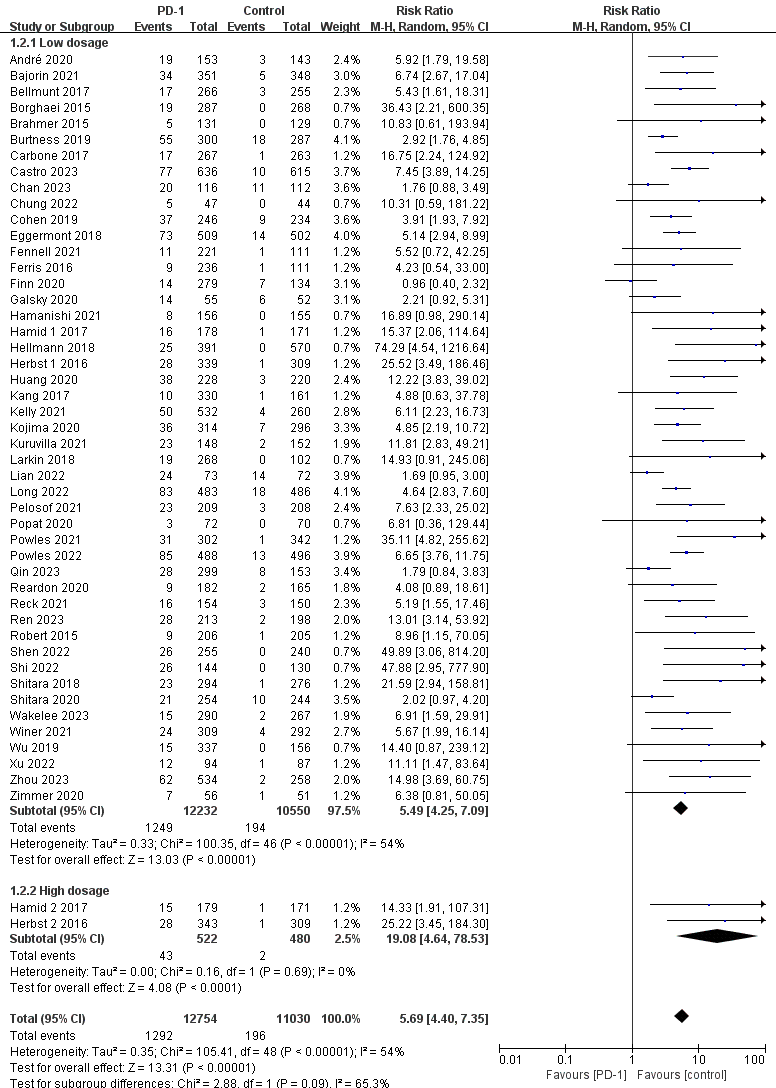
**

**Figure S12.** Forest plots of the relative risks of hypothyroidism related to different doses of PD-1 inhibitors.

**
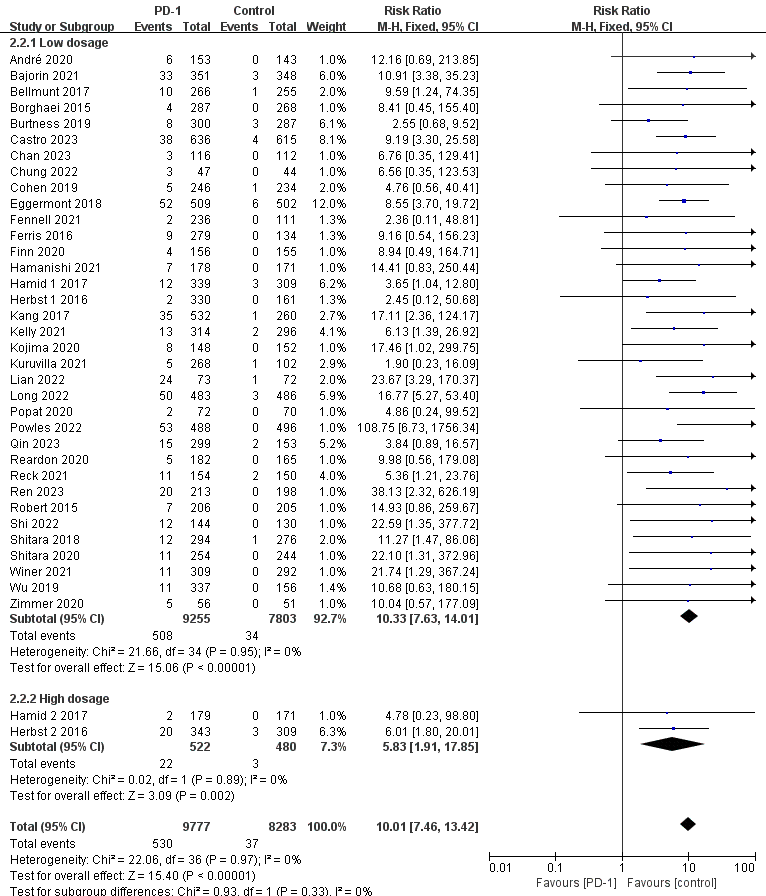
**

**Figure S13.** Forest plots of the relative risks of hyperthyroidism related to different doses of PD-1 inhibitors.

**
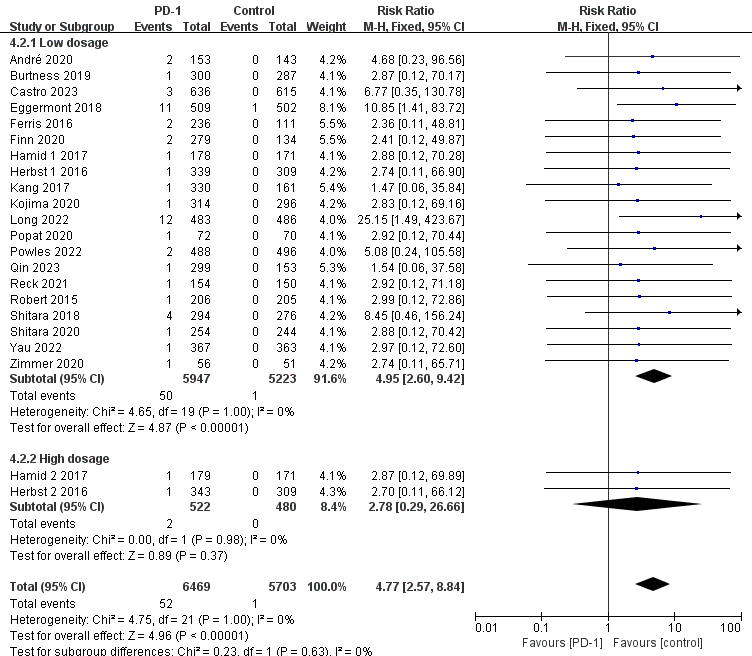
**

**Figure S14.** Forest plots of the relative risks of hypophysitis related to different doses of PD-1 inhibitors.

**
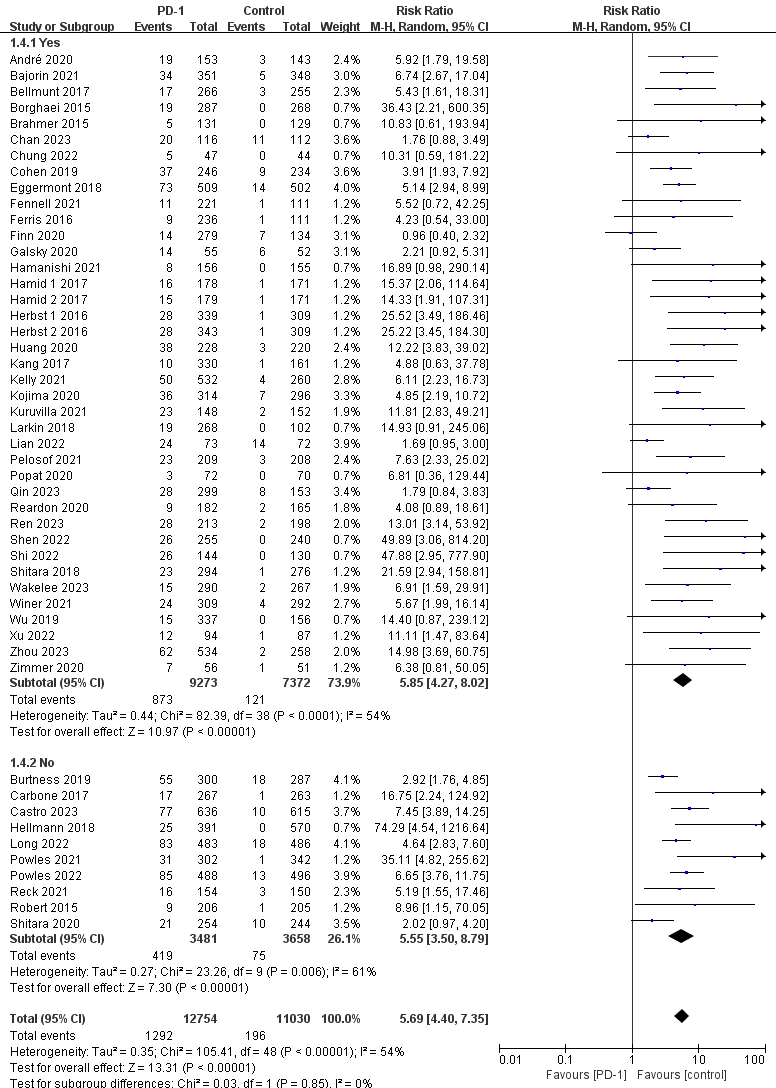
**

**Figure S15.** Forest plots of the relative risks of hypothyroidism related to previous treatments.

**
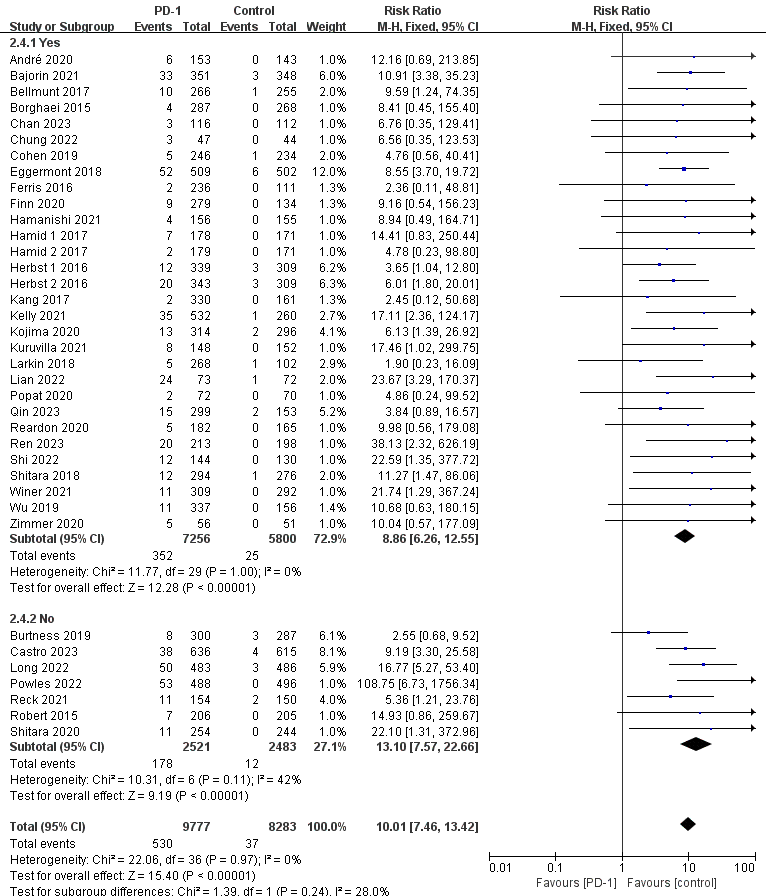
**

**Figure S16.** Forest plots of the relative risks of hyperthyroidism related to previous treatments.

**
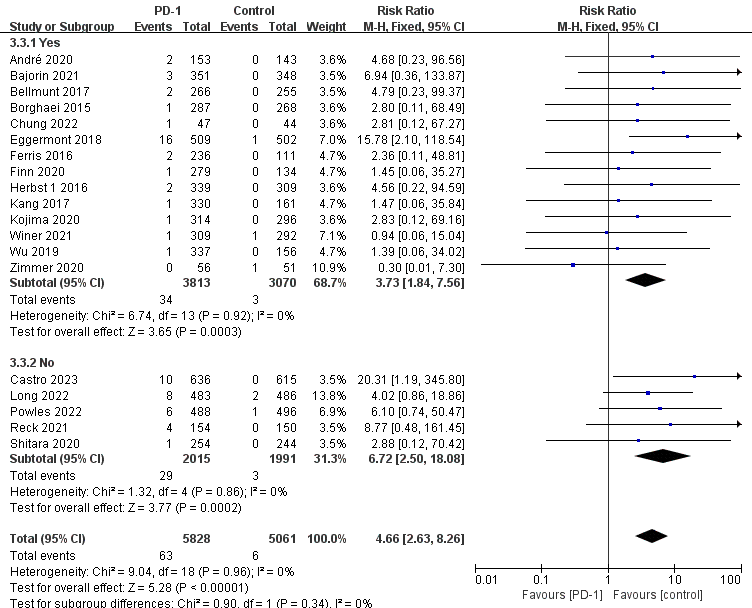
**

**Figure S17.** Forest plots of the relative risks of thyroiditis related to previous treatments.

**
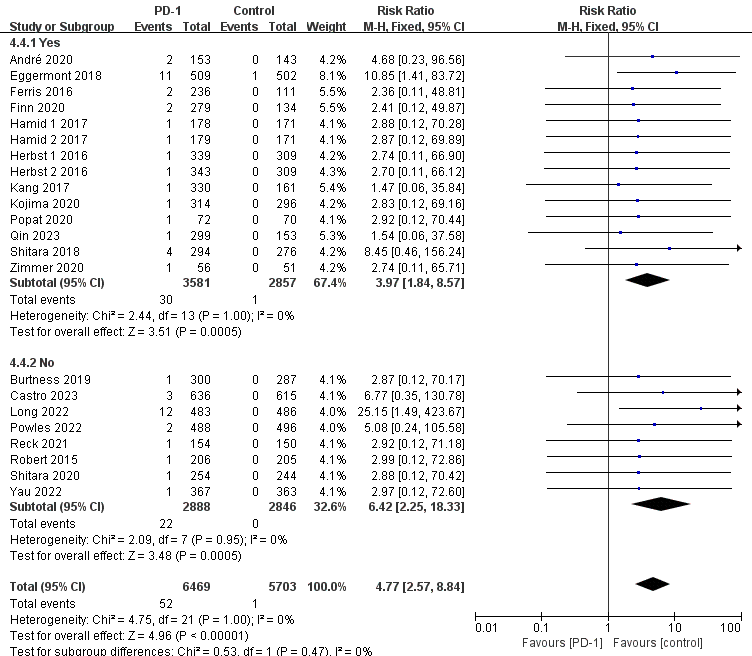
**

**Figure S18.** Forest plots of the relative risks of hypophysitis related to previous treatments.

**
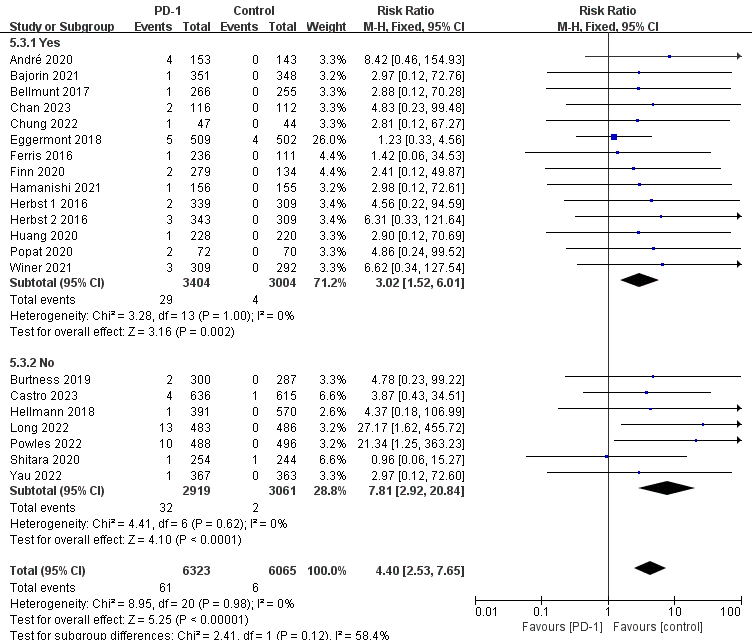
**

**Figure S19.** Forest plots of the relative risks of adrenal insufficiency related to previous treatments.

**
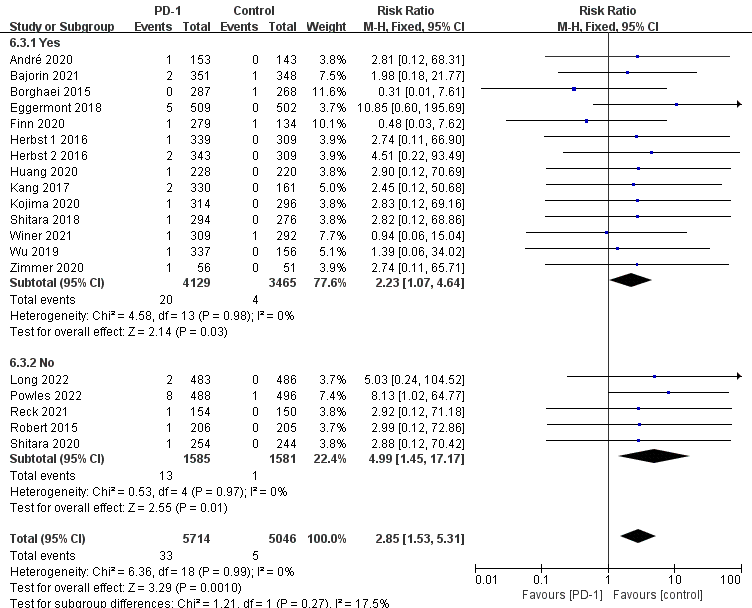
**

**Figure S20.** Forest plots of the relative risks of diabetes mellitus related to previous treatments.
